# The Importance of Livestock Demography and Infrastructure in Driving Foot and Mouth Disease Dynamics

**DOI:** 10.3390/life12101604

**Published:** 2022-10-14

**Authors:** Kendra Gilbertson, Peter Brommesson, Amanda Minter, Clayton Hallman, Ryan S. Miller, Katie Portacci, Stefan Sellman, Michael J. Tildesley, Colleen T. Webb, Tom Lindström, Lindsay M. Beck-Johnson

**Affiliations:** 1 Department of Biology, Colorado State University, 1878 Campus Delivery, Fort Collins, CO 80523, USA; 2 Department of Physics, Chemistry and Biology, Division of Theoretical Biology, Linköping University, 581 83 Linköping, Sweden; 3 Zeeman Institute for Systems Biology and Infectious Disease Epidemiology Research (SBIDER), School of Life Sciences and Mathematics Institute, University of Warwick, Coventry CV4 7AL, UK; 4 USDA APHIS Veterinary Services, Center for Epidemiology and Animal Health, Fort Collins, CO 80526, USA

**Keywords:** foot and mouth disease, livestock demography, model assumptions, cattle shipment networks, outbreak simulation

## Abstract

Transboundary animal diseases, such as foot and mouth disease (FMD) pose a significant and ongoing threat to global food security. Such diseases can produce large, spatially complex outbreaks. Mathematical models are often used to understand the spatio-temporal dynamics and create response plans for possible disease introductions. Model assumptions regarding transmission behavior of premises and movement patterns of livestock directly impact our understanding of the ecological drivers of outbreaks and how to best control them. Here, we investigate the impact that these assumptions have on model predictions of FMD outbreaks in the U.S. using models of livestock shipment networks and disease spread. We explore the impact of changing assumptions about premises transmission behavior, both by including within-herd dynamics, and by accounting for premises type and increasing the accuracy of shipment predictions. We find that the impact these assumptions have on outbreak predictions is less than the impact of the underlying livestock demography, but that they are important for investigating some response objectives, such as the impact on trade. These results suggest that demography is a key ecological driver of outbreaks and is critical for making robust predictions but that understanding management objectives is also important when making choices about model assumptions.

## 1. Introduction

Transboundary animal diseases (TAD) can significantly impact economies and food security worldwide, and require regional or international efforts to contain [1]. They are economically costly via direct animal loss, loss of animal production, market shocks and drop in consumer demand, export trade restrictions, prevention and control costs, impacts on human health for zoonoses or spillover to other species, and disruption of the food supply [1]. TAD examples include African swine fever, classical swine fever, foot-and-mouth disease, several strains of avian influenza, and others [2]. Global dynamics of these pathogens will continue to change as a result of globalization, changing agricultural ecosystems, ecosystem incursion, and climate change [3,4].

One TAD of particular interest globally is foot-and-mouth disease (FMD), a highly transmissible viral infection primarily affecting divided hoofed animals, including cattle, pigs, sheep, and goats. The disease is generally not fatal in adult animals but causes production loss, and lingering infection may cause further outbreaks later on [5]. Outbreaks have cost countries billions of dollars in direct and indirect losses, and the most severe outbreak in the United States (U.S.), in 1929, spread to 22 states and over 172,000 livestock animals were culled [5,6,7]. Given the cost of an outbreak, FMD cases trigger the highest level of restrictions on international trade of all TADs [1].

The U.S. is currently FMD free, and the livestock population remains entirely susceptible [5]. The largest livestock industry susceptible to FMD in the U.S. is the cattle industry. The U.S. cattle industry is large and has specific spatial structuring due to the infrastructure, production practices and resources [8,9,10]. The cattle industry is connected across the entire continental U.S. by a highly connected shipment network [8,11]. Shipments are important in outbreaks of FMD because they can move infection into areas that were not previously infected [12,13]. Because of the scale of the U.S. livestock industry, a potential FMD outbreak could have serious economic implications and there is a need for robust preparedness and response plans.

Many countries that are currently FMD free rely on model simulations of FMD outbreaks to evaluate control measures and factors affecting disease spread in order to inform preparedness and planning. A number of FMD simulation models are reviewed in Webb et al. [14]. These models fall roughly into two types of approaches to capture the spatio-temporal patterns of pathogen spread. First, some models utilize specific and detailed pathways of transmission. These models are useful for investigating specific scenarios but can be difficult to scale up to large spatial scales due to the increased computational burden inherent in tracking many detailed contacts. Second, other models use phenomenological spatial kernels that represent a mixture of specific transmission pathways and thus are more computationally efficient and can make inference at large spatial scales. Some models include a combination of these approaches. These two approaches represent different conceptualizations of the detailed ecological transmission mechanisms of FMD and utilize different practical solutions to the tradeoff between capturing transmission accurately enough and computational efficiency. However, all disease models must make assumptions and choices in what aspects of the ecological reality are captured, resulting in caveats or uncertainty in model structure. In this study, our goal is to understand the impact of capturing three drivers, commonly thought to be important, in order better understand how uncertainty in these drivers affects our general understanding of the system and decision support for FMD preparedness and planning [14]. For instance, assumptions about within-herd transmission may scale up to impact broader spatial scale transmission dynamics. Within a modeling framework, evaluating the importance of within-herd transmission may mean quantifying of outbreak behavior when herds become infected over time (hereafter referred to as partial transition of disease states), rather than an assumption that 100 percent of the herd was infected simultaneously. We are also interested heterogeneity in transmission behavior, for example among different premises types (e.g., farms, markets, feedlots). Finally, variation in long distance transmission events is also of interest. Long distance transmission is associated with livestock movement, so from the modeling perspective the accuracy with which livestock movements are implemented may be important.

To explore these questions about how modeling assumptions impact predictions of FMD outbreaks, we focus on the U.S. cattle industry. The large range in premises sizes, the spatial structuring, and the importance of the shipment network in connecting the industry make this an ideal system to explore the impact of ecological drivers of FMD. The United States Disease Outbreak Simulation (USDOS) is a stochastic simulation model that includes both local and shipment-based disease transmission [10,15]. Because livestock shipments in the U.S. are not tracked unless they cross a state line, the shipment-based transmission in USDOS is predicted by the United States Animal Movement Model (USAMM). USAMM estimates the full U.S. cattle shipment network based on partially observed data [11,16]. In this study, we add additional detail to these two models in order to asses the impact of structural uncertainty while preserving the ability to simulate FMD at the U.S. national scale. In USDOS we expand the model to allow for partial transition of disease states and explore the impact of transmission behavior of livestock markets. In USAMM we explore the impact of adding industry covariates on predictions of the livestock shipment network and on predictions of disease spread by USDOS. We compared model results predicted with and without the structural expansions to understand the impact on predicted outbreak metrics because changes to outbreak size, duration and transmission behavior can impact the decisions made about an outbreak. The results of this study will inform FMD and TAD modeling efforts by highlighting the importance of understanding how modeling assumptions about the ecological drivers in the system can impact the predicted results and will help researchers support decision makers by tailoring the model assumptions to the management objectives.

## 2. Methods

### 2.1. USDOS Version 2.1

USDOS is a national model for predicting disease spread through both local and long-distance shipment-based transmission. Local spread consists of aerosol, fence-line contact, or fomite spread between premises and is captured by a spatial kernel, while long-distance spread occurs during shipments between premises in any two counties in the contiguous U.S. [10,15]. At the beginning of a simulation, each premises in the contiguous U.S. is given a disease status, which is tracked throughout the outbreak. Premises disease statuses are susceptible to infection, exposed and pre-infectious, infectious, or immune (e.g., due to recovery or vaccination). Here, we introduce USDOSv2.1, which incorporates more detail in premises partial transition of disease states, heterogeneous transmission behavior of premises, and livestock movements.

### 2.2. Premises Location and Size

There are three types of premises in the demography: general cattle premises, that are further separated into beef or dairy; feedlots; and markets. Because U.S. cattle premises locations are not publicly accessible, premises data for the general cattle premises and feedlots were generated by the Farm Location and Agricultural Production Simulator (FLAPS) [17,18]. FLAPS generates predicted premises locations and number of cattle per premises in the U.S. The number of premises and the sizes of those premises in each county are based on the totals in the National Agriculture Statistics Survey (NASS) 2012 census data and the NASS cattle inventory survey from July of 2017 [10,17,19]. The locations of premises in counties are estimated by FLAPS based on environmental factors important for the presence of livestock [18]. The data sets to independently validate FLAPS simulations do not exist, therefore, Burdett et al. [18] performed verification analyses that showed good predictive performance by the model. We used 10 FLAPS realizations of cattle demographic data to reflect the uncertainty in the FLAPS estimates [10].

A list of cattle market locations was generated by Carroll and Bansal [20] from several publicly available sources; specifically USDA APHIS Federally Approved Market List, USDA GIPSA, USDA Agricultural Marketing Service (AMS), and Livestock Market Association (LMA) [20,21,22,23]. We updated and consolidated the list to remove duplicate entries and geocoded the location information to the county level. Further refinements of the list and the locations of the markets were identified by investigating cases where the consolidation assignments on the list did not match up with the geocoded county information (i.e., cases where entries on the list were previously assigned to be the same market were geocoded to different counties, or cases where entries assigned to be different markets were geocoded to the same county). AMS provides data on annual cattle sales totals for some of the markets on the list. We used these data to inform a spatial model that estimated the county-level values for total cattle sales in each county containing a market. These estimates were used to inform the market sizes. The market locations and sizes were combined with each of the 10 FLAPS realizations to create the cattle demography data used here.

### 2.3. Local Disease Transmission Kernel

Local disease transmission encompasses all transmission events that are not shipments of infected cattle. Here we use a distance-dependent transmission kernel to describe local transmission. This type of kernel provides an accurate fit to FMD outbreak data [12,24,25,26] and is a commonly used method for capturing local transmission in FMD models [27]. Here, the probability of disease transmission from infectious premises *i* to susceptible premises *j* on a given day is described as in Tsao et al [10].

(1) $$\begin{array}{c} 1-\exp(-a_{c}h_{i}b_{c}h_{j}K\left(d_{i,j}\right)),\; \end{array}$$

where $h_{i}$ and $h_{j}$ represent the size of infectious and susceptible herds, $a_{c}$ is the cattle transmissibility parameter, and $b_{c}$ is the cattle susceptibility parameter. *K* is the distance-dependent kernel function where $d_{i,j}$ is the shortest linear distance between *i* and *j*, defined as

(2) $$\begin{array}{c} K\left(d_{i,j}\right)=\frac{k_{1}}{1+{\left(\frac{d_{i,j}}{k_{2}}\right)}^{k_{3}}},\; \end{array}$$

where $k_{2}$ is the scale parameter and $k_{3}$ is the shape parameter. The $k_{1}$ parameter is the normalizing constant that scales the function so that

(3) $$\begin{array}{c} \int_{0}^{\infty }2\pi rK\left(r\right)dr=1,\; \end{array}$$

where *r* is a dummy variable for $d_{i,j}$, the distance between premises *i* and *j*. At a small distance, transmission is consistently high and decays as the distance increases. The power-law kernel function (Equation (2)) has been fit to several FMD outbreak data sets and used in previously published versions of the USDOS model and other FMD models [10,12,15,28,29].

The local transmission parameters (Table S2) were fit using a maximum likelihood approach to the first 30 days after the official recognition of 2001 UK outbreak data, which is a time period where the outbreak was growing and control was consistent [10]. The parameters were fit in meters to match with the units of distance used in USDOS. The best fit parameters give mean outbreak sizes of approximately 1600 infected premises (95% confidence interval: 1000–2800 infected premises, compared with 2026 infected premises in the UK outbreak [12]) [10]. Because there has not been a recent FMD outbreak in the U.S., we ran a sensitivity analysis on the local transmission kernels that encompassed wide range of parameter values [10]. The results of this sensitivity analysis, presented in Tsao et al. [10] show that USDOS outbreak metrics are less sensitive to these parameters than to other aspects of the system.

### 2.4. Partial Transition of Disease States

In USDOSv2.0 [10], premises are classified as either susceptible, exposed, infectious or immune. When within-herd dynamics are not considered (as in USDOS2.0, [10]), the rate at which an infectious premises *i* infects a susceptible premises *j* is given by,

(4) $$rate_{\left(i,j\right)}=\left(\left[N_{\left(beef,j\right)}^{p_{beef}}\right]S_{beef}+\left[N_{\left(dairy,j\right)}^{p_{dairy}}\right]S_{dairy}\right)\times \left(\left[N_{\left(beef,i\right)}^{q_{beef}}\right]T_{beef}+\left[N_{\left(dairy,i\right)}^{q_{dairy}}\right]T_{dairy}\right)\times K\left(d_{ij}\right)$$

where $N_{\left(b,i\right)}$ is the number of individuals of species *b* on premises *i*, $S_{b}$ and $T_{b}$ are the susceptibility and transmissibility measures for premises of type *b* and $p_{b}$, $q_{b}$ are power law parameters accounting for a non-linear increase in susceptibility and transmissibility as animal numbers on a premises increase. Infection spreads between premises via the transmission kernel *K* according to the distance between premises *i* and *j*, $d_{\left(i,j\right)}$.

For USDOSv2.1, we extended the USDOSv2.0 framework outlined above to incorporate within-herd dynamics such that infectiousness is dependent upon how many animals on the premises are infectious at time *t*. Therefore, instead of using a fixed premises size $N_{\left(beef,i\right)}$ as in USDOSv2.0, we used the number of infectious animals at time *t*, $I{\left(t\right)}_{\left(beef,i\right)}$. The relevant equations and technical details are given in Supplementary Section S1.1. In order to determine the best fit parameters for the partial transition function I(t), we minimized the sum of the squared difference between “data” (mean of 1000 simulations from an FMD within-herd model [30]) and the predicted values from the partial transition function (Table 1). The partial transition function describes how many animals are infectious at time *t* after infection has entered the premises and the latency period has passed. With some mathematical manipulation, the partial transition function can be expressed as a function of time, *t*, describing the proportion of the premises’ population that is infectious after *t* days such that, regardless of premises size, the proportion will always be the same for each value of *t* and can be pre-calculated in USDOSv2.1 (Figure S2, details in Supplementary Section S1.1). An important issue is how long the within-herd model dynamics should be considered before it is practically the same as assuming 100 percent of the herd is infected. We considered an infectious period cutoff of 15 days, 20 days, and 30 days to examine computing time and how much of the infection each period captured. For additional details on the partial transition function, fits, and parameters, see Supplementary Section S1.1.

### 2.5. Accuracy of Livestock Movement

Livestock shipment data is not routinely collected in the U.S., and consequently very little official data is available to utilize in a livestock disease simulation. Therefore, animal movements in USDOS are dynamically simulated during the outbreak using the United States Animal Movement Model (USAMM). Cattle shipped interstate in the U.S. are required to be accompanied by a certificate of veterinary inspection (ICVI) to ensure that the animals are healthy prior to being shipped. Paper records of the ICVIs are kept by the origin and destination state authorities, and among other things, these records lists the shipment date as well as the origin and destination counties of the shipment. In an effort to provide a first detailed picture of the U.S. cattle trade network, the authors of Buhnerkempe et al. [8] collected a 10% sample of the 2009 export ICVIs of each state of the conterminous U.S., excluding New Jersey, and compiled it into an electronic data set of interstate beef and dairy movements. This data set remains the most geographically comprehensive cattle shipment data set available, and USAMM has been constructed specifically to scale up this limited sample of inter-state shipment data to complete nation-wide cattle movement networks consisting of both inter- and intra-state shipments. Two versions of USAMM have been published previously—USAMMv1.0 [11] and and USAMMv2.0 [16]. In USAMMv1.0, shipments are modeled using a state-level distance dependence together with state-level covariates consisting of historical inflow data, and county-level weights scaling linearly with the total number of premises in the county. USAMMv2.0 improves on the first version in several ways and is described briefly below. Here we introduce an extension of USAMMv2.0, which we refer to as USAMMv2.1, that includes the effect of county-level covariates related to the cattle industry, as well as premises type-specific county-level weights based on non-linear herd-size dependent scaling of the farms, feedlots and markets in the county. In order to evaluate the effect of cattle related infrastructure on the transmission of FMD we simulate the shipments within USDOS with both USAMMv2.0 and USAMMv2.1 and analyze the relative importance and the improvement in fit using a model selection framework. We briefly describe these developments in the following sections but additional details of the USAMMv2.1 development, testing, and implementation can be found in Supplementary Sections S1.2–S1.8.

#### 2.5.1. USAMM Version 2.0

In USAMMv2.1, we built on the earlier USAMMv2.0 movement model of Brommesson et al. [16] to improve the previous statistical model for the probability of observing a set of interstate cattle shipments given by the 2009 ICVI data. In this section, we describe this previous model, and in the next section outline the changes that were made to it in the present work. Briefly, it was assumed in USAMMv2.0 that within each state shipments arose from each single premises as a Poisson process with the premises-level shipment rate $\lambda_{u}^{*}$. Given this assumption, the collective rate with which shipments arose from each origin county was simply the number of premises in the county multiplied by $\lambda_{u(\omega )}^{*}$. The shipments that arose from an origin county were then distributed among all possible destination counties, i.e., all other counties in the U.S. This last step was achieved by assigning each destination county a weight based on an attraction parameter of the destination state, as well as a function of the distance between the origin and destination counties and two origin state-level parameters. To determine the destination county weight, each state was associated with a parameter controlling the propensity for a single premises in the state to attract shipments. When scaled up by the number of premises in a county, this gave a total measure of the entire county’s capacity to attract shipments based on the size of its population of premises. The two origin state-level parameters occur because each state was associated with a monotonically decreasing distance kernel function which gave a distance dependence component to the county–to–county shipment probability. The spatial kernel is plateau-shaped at short distances and has a fat tail describing the probability of long distance shipments. USAMMv2.0 included a component of seasonality with parameters being defined for each quarter of the year. A full mathematical description and details on parameter estimation are given in Supplementary Section S1.2.

#### 2.5.2. USAMM Version 2.1

In USAMMv2.0, each premises is given equal weight and county-level shipping differs simply due to the number of premises in the respective county. Analyses of other systems have however revealed that both the number of incoming and outgoing shipments vary with the type and herd size of the premises [31]. In USAMMv2.1, we therefore relaxed the assumption that all premises send and receive the same number of shipments and modeled the contribution of each individual premises as a function of its herd size (i.e., number of animals) and type (i.e., farm, feedlot, or market). For markets, we used the total yearly volume (measured in head) as herd size. We also decoupled the probability that a premises sends a shipment from the probability that it receives a shipment within a county. We assumed that the relationships between herd size and shipment probability can be nonlinear and model them in the form of a set of power laws with parameters specific to premises type, quarter, and direction. In addition to the differentiation between premises types and premises of different size, we introduced two county-level weighting parameters that independently impact the probability of sending a shipment and the probability of receiving a shipment. These weights depend upon county-level livestock industry covariates, specifically the NASS categories Operations with Sales, Total Sales (in head) [32] as well as a metric describing the closeness to slaughter capacity, Slaughter Connectivity. These particular categories were chosen based preliminary correlational analysis with the number of incoming and outgoing ICVI shipments per county. Despite the new additions, the likelihood function of USAMMv2.1 is equivalent to that defined for USAMMv2.0 with redefinition of some parameters so that the new model allows for the inclusion of county and premises level heterogeneities. However it is also possible for the model to disregard one or both of them for certain parameter values. The latter is equivalent to the base model in Brommesson et al. [16] which can be viewed as a special case of USAMMv2.1 where each county has an effect of industry covariates equal to one (i.e., no effect of covariates), and where every premises has equal weight regardless of size and type. A full mathematical description and details on parameter estimation are given in Supplementary Section S1.3.

#### 2.5.3. USAMM Model Selection

To determine if including industry covariates and information about premises type and size provides better fit to data, we compared USAMMv2.1 to a simpler version where all parameters modeling the effect of industry covariates and premises characteristics were fixed at a value that indicated no effect. We refer to this simplified version as “simple” and the full USAMMv2.1 model presented above as “refined”. We used Widely Applicable Information Criterion (WAIC) [33] to indicate which model was the most parsimonious for the focal data. WAIC is derived from estimating the out-of-sample predictive accuracy using within-sample data and adjusting the introduced overestimation by a penalty term estimating the number of effective parameters [34]. WAIC uses log pointwise posterior predictive density as a measure of fit and (as proposed in Gelman et al. [34]) the posterior sample variance of the log predictive density as penalty term. This pointwise approach is more fully Bayesian and captures the posterior uncertainty better than other information criteria [34], which makes WAIC the preferred choice.

### 2.6. USDOS Model Scenarios

We explored the effects of modeling assumptions by performing a structural sensitivity analysis with USDOS. The structural sensitivity analysis was done by running USDOS model scenarios that systematically included and excluded model components that corresponded to partial transition of disease states, and the accuracy of livestock movements. The changes in USDOS model predictions observed between the different structural sensitivity scenarios were measured using outbreak metrics, which are described in the following section. The scenarios began with all premises and counties in the contiguous U.S. susceptible except for one randomly selected exposed premises located in the seed county. Each of the 3049 contiguous counties were seeded 100 times (10 times per FLAPS realization), and within the county a random seed premises was selected each time. Simulations continued until the outbreak died out (zero exposed or infected premises) or 365 days had passed, whichever happened first. A complete national-scale run of each scenario results in 304,900 simulations of USDOSv2.1 in order to estimate the characteristics of FMD outbreaks beginning in any county in the contiguous U.S. [10].

The scenarios used to explore structural sensitivity to partial transition of disease states were:

- Base scenario (no control measures in place) without partial transition of disease states;
- Base scenario with partial transition of disease states, and an infectious period cut-off of 15 days;
- Base scenario with partial transition of disease states, and an infectious period cut-off of 20 days;
- Base scenario with partial transition of disease states, and an infectious period cut-off of 30 days.

All partial transition scenarios were run with the refined USAMM model as that was the preferred model in USAMM model selection. We compared base runs (no control measures) with an infectious period cutoff of 15 days, 20 days, and 30 days to examine computing time and the effect of cutoff time on capturing disease dynamics (see Supplementary Section S1.1). We also compared the results of our partial transition base run with 20-day cutoff to our base run without partial transition. We used the 20 day cutoff for this comparison because it was long enough to capture the infection dynamics but short enough to keep computational time down (see Supplementary Section S2.1 for additional details). Using a base run demonstrates the effect of partial transition on outbreaks without introducing confounding effects from control measures. The default infectious period for a base run without partial transition is seven days.

The scenarios exploring the accuracy of livestock movements were:

- Base scenario: no control measures in place.
- Infected premises (IP) cull and 3 km ring vaccination scenario: infected and reported premises (IP) culling and ring vaccination, which is a solid circle centered on the IP, with a radius of 3 km. Animal shipment is banned at the state-level with 75% effectiveness.
- IP cull and 10 km ring vaccination scenario: IP culling and ring vaccination with a radius of 10 km. Animal shipment is banned at the state-level with 75% effectiveness.
- IP cull and dangerous contact (DC) vaccination: IP culling and vaccination of DCs, which are premises with an epidemiological link to an IP. Animal shipment is banned at the state-level with 75% effectiveness.

All base and control scenarios were run with the simple and refined USAMM model, without partial transition. We compared the results of our base runs without partial transition with the simple and refined USAMM models to see what, if any, effect the accuracy of livestock shipments had on our simulations without any possible confounding effects from the within-herd dynamics or control measures. The control scenarios chosen for our analysis were based on strategies analyzed as potential alternative controls for previous FMD outbreaks [12,35,36,37], and in Tsao et al. [10]. Additionally, the preferred control scenarios in the U.S. are strategies that include vaccination [38], so all the control scenarios used here include vaccination; however, these scenarios should not be considered policy. Comparing the differences between control scenarios run with simple and refined USAMM models allowed us to explore whether predictions about outbreak control shifted in response to changes in livestock shipment accuracy. Shipment bans are implemented after the first infected premises is reported within a state and represent state-wide prohibition on cattle shipments. We simulated this ban as being 75% effective. Tsao et al. [10] includes additional information on the control parameters used in this model.

### 2.7. Outbreak Metrics

All metrics were calculated in post-processing of simulation runs using R versions 3.6.3–4.1.1 [39]. We chose the following six metrics to evaluate the results of our simulated outbreaks:

1. Number of premises infected: the total number of infected and reported premises.
2. Number of infected counties: the total number of counties that infection spreads to when infection is seeded in that county.
3. Outbreak duration: the number of days between the initial seed infection until there are no more infected premises or 365 days, whichever occurs first.
4. Outbreak take-off (sensitivity analysis only): the probability that over 5000 premises will become infected during the outbreak [10].
5. Outbreak fade-out (sensitivity analysis only): the probability that between one and 5000 premises will become infected during the outbreak, and that duration will be shorter than 365 days [10].
6. Proportion local transmission: the proportion of non-shipment transmission within each county compared to total transmission (shipment and local) within the county.

These outbreak metrics provide a thorough understanding of the outbreak size and characteristics, and include metrics that are commonly used to quantify both observed and model predicted FMD outbreaks [10,12,35,36,40,41]. For each outbreak metric we calculated both the median and upper 97.5th percentile value for each county aggregated across all simulations within a scenario. We interpret the median as our usual expectation of what might happen if infection was introduced, but we generally see that the median was very low. However, very large outbreaks can occur, so we used the upper 97.5th percentile to capture lower likelihood, but high risk, scenarios. The results we present represent what we expect to see from each county across all simulations in order to give a more robust understanding of the outbreak patterns.

### 2.8. Sensitivity Analysis

The sensitivity analysis allowed us to examine which parameters are important for driving the outcomes of our simulations distinct from the disease dynamics. For the sake of computational time, the sensitivity analysis was run on a subset of 78 counties selected using stratified random sampling based on county level characteristics: total in shipments, total out shipments, premises clustering quantified by Ripley’s K, premises density, and number of large premises. Eight additional counties were included to enhance geographic range or drawn from a list of six counties important to the cattle industry (see Tsao et al. [10] for details). We also recorded the seed premises size. This variation in county- and premises-level characteristics helped us to understand if our results are dependent on where in the country the outbreak begins.

We used Latin hypercube (LHC) sampling to generate values for two parameters: shipment scale and market within scale. Shipment scale adjusts the number of shipments USAMM predicts, which is a different type of test of how the accuracy of livestock movements impacts inference because it allows for over- and under-prediction. Market within scale adjusts the amount of transmission occurring at markets, which allows us to test how differences in transmission between farms/feedlots and markets impact inference. We generated 100 combinations of the two parameters. We chose not to include disease parameters in the sensitivity analysis as we have previously found that outbreak metrics are more sensitive to demography than disease parameters [10]. Hence, we focused on the variation contributed by our county set, seed premises size, and two parameters that impact the accuracy of livestock movements and transmission differences among premises types. Each of the 100 parameter combinations were simulated 100 times for a total of 10,000 simulations for each of the 86 counties. All simulations included partial transition and the refined version of USAMM.

Because our model results were monotonic but not linear, we first used a partial-rank correlation coefficients (PRCC) analysis to estimate the relative importance of each covariate to the outbreak metrics [42]. We then used a linear regression model which provides greater accuracy [43] and allows us to explore two-way interactions between covariates [10,15]. Understanding the effect of interactions between covariates is important because many of the covariates in this system are not independent of each other and some, such as the demographic characteristics, have uncertainty in them. Understanding how multiple attributes of the system interact in terms of disease outbreaks can help identify counties with higher risk. Regression has a stricter assumption than PRCC, in that the relationship between outbreak metrics and covariates is linear. However, regression is fairly robust to this assumption. In order to assess the appropriateness of using regression to understand interaction, we first performed linear regression with no interaction and compared the results to those of the PRCC analysis. Based on the similarity between these results, we felt comfortable proceeding with the full two-way interaction regression model, despite the violation of linearity within our data [10,15].

## 3. Results

### 3.1. Partial Transition

We found that a partial transition maximum infectious period cut-off of 15 days was too short to fully capture the outbreak dynamics and led to outbreak predictions that were substantially different to those predicted by scenarios run with a cut-offs of 20 and 30 days (Figures S4–S6). The 20 and 30 day cut-off scenarios predicted similar results. The addition of partial transition more than doubled the computing time for USDOS, with longer maximum infectious period cut-offs leading to longer run times (Table S3). Because the 20 day maximum infectious period cut-off accurately captured the outbreak dynamics and reduces computational time compared with the 30 day cut-off, assigned 20 days as the default maximum infectious period length in USDOS (additional details in Supplementary Section S2.1). The following results that include partial transition all use this default value.

**Figure 1:**
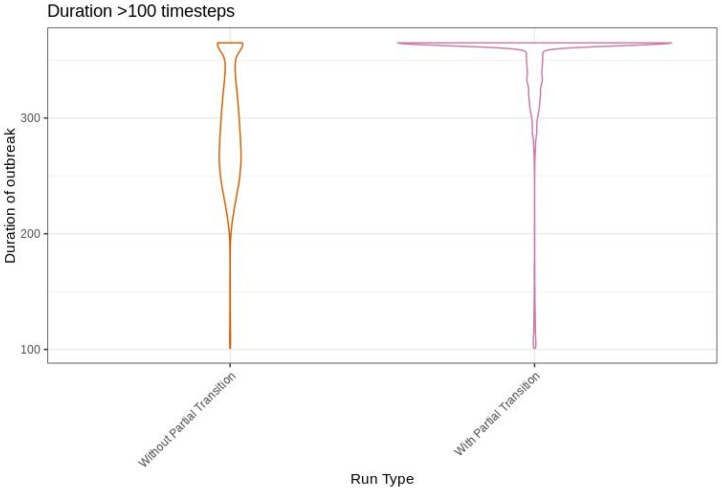
Duration of outbreak simulations with partial transition off (orange, left) and on (pink, right) for outbreaks lasting longer than 100 days.

We compared the outbreak metrics from the base scenario without partial transition with those from the base scenario with partial transition. Duration was the outbreak metric most affected by including partial transition in the model. Including partial transition in USDOS increases the length of time premises are infectious which leads to an increase in the total duration of an outbreak. Comparing the median and upper 97.5th percentile of outbreaks, we see that duration is longer for the scenario with partial transition by 5 days for the median and 100 days for the upper 97.5th percentile (Table S4). The increase in duration can be clearly seen by comparing the distributions of the 97.th percentile of outbreaks, where far more outbreaks reach the 365 day maximum duration with the partial transition scenario (Figure 1). Spatially, we see the largest effect of duration on the median outbreaks, with an increase in spatial variation in outbreak length and an overall increase in predicted duration (Figure S7). The dominate spatial patterns predicted with and without partial transition for the largest outbreaks (upper 97.5th percentile) are the same but including partial transition increases the amount of area predicted to have the longest outbreaks (Figure S7).

**Figure 2:**
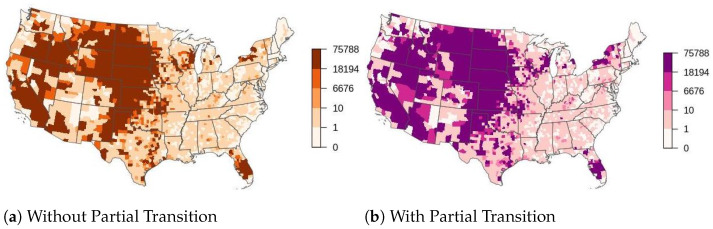
Counties colored by the number of premises infected for the upper 97.5th percentile of runs without partial transition (**a**), and with partial transition (**b**). It is an aggregation across all simulations of the total number of premises infected when the outbreak is seeded in that county.

We also observed that for the upper 97.5th percentile of outbreaks, the base scenario with partial transition predicted larger outbreaks measured by the of number of premises and counties infected than the base scenario without partial transition (Table S4). However, the predicted median behavior of the scenarios with and without partial transition for number of premises and counties infected were the same (Table S4). In comparing the spatial patterns of the upper 97.th percentile of outbreaks we see that outbreaks seeded in counties in Idaho, Montana, Nevada, Arizona, Minnesota, Iowa, and New York resulted in larger outbreaks with partial transition than without partial transition (Figure 2 and Figure S9). However, the geographic pattern remained consistent with and without partial transition; large outbreaks were still produced when disease was seeded along the west coast and a corridor along the mountain west and central plains down into Texas. The Midwest, East Coast, and Southeast generally produced smaller outbreaks with exceptions for New York state and central Florida. The predicted spatial patterns of the median outbreak patterns are much more similar for the metrics of outbreak size, with only a few counties in California, South Dakota and Nebraska having higher predicted outbreak size for the scenario with partial transition (Figures S8 and S9).

### 3.2. USAMM Simple and Refined Versions for Disease Transmission Type

Model selection with WAIC between simple and refined USAMM showed scores strongly in favor of the refined models for both beef (ΔWAIC= 21,895) and dairy (ΔWAIC=3195) shipments (Table 2). This indicated that the additional components of the refined version added informative value and promoted a better description of the data. Further, posterior estimates indicated a pronounced effect of premises size and type as well as industry covariates (Figure S10). The effect of including the additional parameters of the refined model was evident in a more pronounced differentiation between counties with high and low degree. This effect was true for both commodities and for both in- and out-degree and is illustrated in Figures S11 and S12.

**Figure 3:**
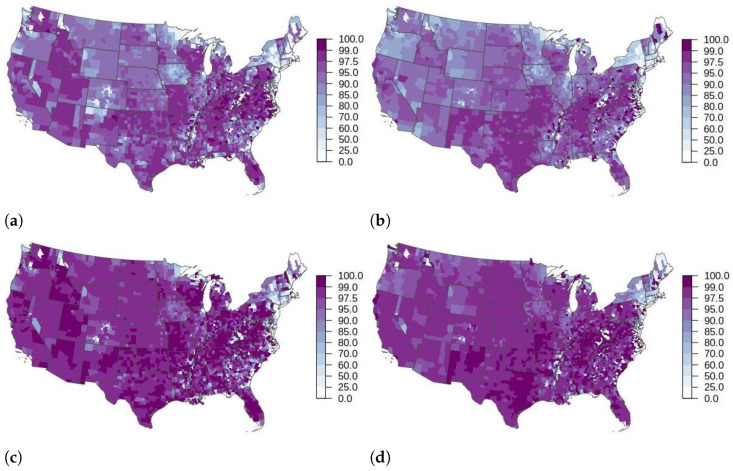
Counties colored by the proportion of local (non-shipment) transmission for base scenarios (**a**,**b**) and cull and 3 km ring vaccination scenarios (**c**,**d**) run with USAMMv2.1 simple (**a**,**c**) and USAMMv2.1 refined (**b**,**d**). (**a**) Base scenario run with USAMM2.1 simple. (**b**) Base scenario run with USAMM2.1 refined. (**c**) IP cull, 3 km ring vacc. scenario run with USAMM2.1 simple. (**d**) IP cull, 3 km ring vacc. scenario run with USAMM2.1 refined.

When comparing the outbreak metrics from the USDOS scenarios run with USAMMv2.1 simple and refined the greatest difference was observed in the proportion of local transmission. The proportion of local transmission was generally higher across the country for scenarios run with USAMMv2.1 simple (Figure 3 and Figure S13). The exceptions to this were several counties in eastern Texas where there was greater proportions of local transmission predicted by scenarios run with USAMMv2.1 refined. This result held across the base scenarios and all of the scenarios with control, though it is more apparent in the base scenarios which do not have shipment bans in place.

The predicted differences between USDOS scenarios run with USAMMv2.1 simple and refined for other outbreak metrics were more subtle, particularly compared with the differences seen above with the addition of partial transition. Nationally, the differences between scenarios run with USAMMv2.1 simple or refined are so small that it would be difficult to separate true differences from stochastic variation (Figures S14 and S15, Table S5). Spatially, there are very few differences between the median predictions from the scenarios using USAMMv2.1 simple and refined but there are more pronounced differences when looking at the upper 97.5th percentile of outbreaks (Figures S16 and S17). As with the results from partial transition, the major spatial patterns do not change. However, scenarios run with USAMMv2.1 refined predicts that the number of counties where the largest outbreaks are predicted to start increases. This is particularly apparent in Montana, Wisconsin and New York (Figures S16 and S17). The control strategy predicted to reduce the outbreak metrics the most was IP cull and DC vaccination. This result was consistent between the scenarios run with the simple and refined models and is also consistent with previously published results from USDOS [10].

### 3.3. Sensitivity Analysis

**Figure 4:**
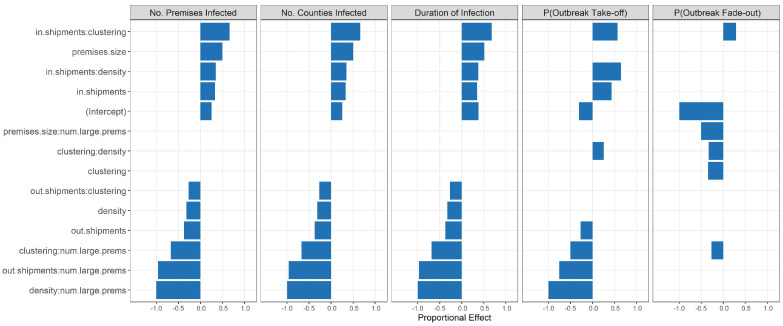
Scaled regression results for two-way interaction models of each outbreak metric including only covariates whose effect size is ≥∣0.25∣. All covariates with an effect size ≥∣0.25∣ are significant at a p=0.001 level.

The results of our sensitivity analysis demonstrated which characteristics were important in determining outbreak metrics across a large number of simulations. Number of premises infected, number of counties infected, duration of infection and probability of outbreak takeoff had virtually identical patterns across covariates (Figure 4 and Figure S18). These four metrics were significantly positively associated with increases in the size of the initial seed premises and in-shipments, along with its interaction with density and clustering. They were significantly negatively associated with increases in density and its interaction with the number of large premises, as well as out-shipments and its interactions with the number of large premises and clustering. In addition, the interaction of clustering and number of large premises also had a significantly negative relationship with these four metrics. The probability of outbreak fade-out had a significant negative association with the interaction of the size of the initial seed premises with the number of large premises. All of the metrics were relatively insensitive to our new parameters, shipment scale and market within scale. All of the covariates that had an effect size of greater, or equal to 0.25 in the regression (*p* ≤ 0.01) were demographic or shipment attributes or interactions between these (Figure 4). The interactions between demographic and shipment covariates show that these attributes are not acting independently in the livestock industry with respect to disease outbreaks.

The shipment scale and market within parameters had a much smaller impact on determining outbreak metrics than the demographic and shipment covariates discussed above (Figure S18). The largest impact was a slight positive association between increasing the shipment scale parameter and the number of premises and counties infected. This suggests that uncertainty in the number of shipments predicted by USAMM is of less importance to outbreak predictions than the structure of the shipment network. The interactions between these parameters and the other covariates in the analysis also had a small impact on the results. The interaction between market within and the seed premises size had a slight positive association with number of premises and counties infected. Together these results suggest that shipment scale and market within are less important than demographic and shipment characteristics in driving outbreak behavior.

## 4. Discussion

With new formulations of USDOS and USAMM, we investigated some of the most important assumptions in models of the spatio-temporal spread of TADs: premises having partial transition of disease states, transmission behavior of premises, and the accuracy of livestock movement, while these may appear to be very technical issues within the realm of modeling, they represent different ideas about the ecology of TADs, what drives the behavior of their spatio-temporal spread, and which drivers are most important. Interestingly, we find that these assumptions have a strong effect on the overall size or intensity of the outbreak, but little impact on the geographic patterns that results from spatio-temporal spread. The behavior of within-herd spread, heterogeneity of transmission from different types of premises, and reasonable variation in long distance transmission via livestock movement all have relatively little impact on the patterns formed by the spatio-temporal dynamics of FMD. Overall, we see that the details of structural assumptions in TAD models as shown here and even parameter values within reason [10], have less impact than the underlying demography of the cattle industry at the scale of the U.S. Premises sizes, density, spatial clustering, and shipping behavior play an outsized role in the spatio-temporal patterns of FMD at the large scale of the U.S. Likely because of this, we also do not see that differences in these types of model structural choices or parameter variation within biological reason (as in Tsao et al. [10]) result in differences in the behavior of different control strategies, e.g., the relative ranking of control strategies remains despite differences in the types of model structures investigated here and across different biologically appropriate parameterizations.

Including partial transition of disease states allowed FMD outbreaks to progress more realistically through a premises. The longer outbreak duration in scenarios that included partial transition are a result of longer infectious times for a given premises, leaving more time for FMD to spread to other susceptible premises. This effect on individual premises scales up to increase the outbreak duration overall and to a smaller increase in outbreak size. However, there was little change geographically in where the largest outbreaks originated. Including partial transition or a within-herd model in a large outbreak model should be considered when predictions about duration and trade restrictions are important. Approximating the impact of within-herd dynamics by increasing the length of time herds are infectious without partial transition would increase the outbreak duration and the size but would likely lead to a major overestimation because the entire herd would be infectious for the infectious period, while the partial transition function considered here is also an approximation of a full within-herd model, it provides a good estimate for how smaller-scale dynamics impact broader outbreak patterns for a fast spreading infection like FMD and does not present the same overestimation problem. The partial transition approximation of within-herd dynamics would not be as appropriate if the disease of interest was slower spreading or had more complex transmission dynamics. In such cases, a full within-herd model may be needed.

We saw little impact of variation in the transmission behavior of premises impacting USDOS predictions. Outbreak metrics were relatively insensitive to the new parameter we introduced to allow greater heterogeneity in transmission between markets and other premises types. Markets are important premises for the mixing and moving of livestock, but the speed of FMD transmission and the number of markets relative to beef and dairy premises in this system accounts for why the within-market transmission behavior does not have much impact on predicted dynamics. The impact on variation in transmission behavior due to differences in the versions of USAMM, one of which included greater premises detail, is discussed in detail below, but it appears that differences in the two versions are due more to the inclusion of industry covariates than the effect of including premises size and type information.

Uncertainty in livestock movement potentially has consequences for predictions of disease spread because differences in connectivity can alter risk of transmission [29]. Data on intrastate shipments in the U.S. are scarce and incomplete at the national-level, making these shipments a key unknown in understanding shipment patterns in the contiguous U.S. In other countries, shipment data may exist, but they have been used from a historic perspective. Thus, the issue of uncertainty in knowing livestock shipments that are relevant to an outbreak is a common one globally. The effects of premises size and type as well as industry covariates, which are contained in the refined version of USAMM, all were estimated to affect shipment rates and provided added value in the accuracy of predictions. We suspect that the information on industry covariates helps to more accurately capture the heterogeneity in county shipment levels. Based on our experience, it is particularly difficult to capture a few important counties that act as "super shippers" with more extreme values of in-shipments and out-shipments. The refined model allows for larger shipping parameters for super shipper counties, leading to more shipment-based disease spread in USDOS. The counties where we see a change in shipment-based spread are in areas known to have the most beef and dairy infrastructure, which is consistent with the super shipper interpretation.

The differences in livestock movement predictions from the two versions of USAMM led to some differences in USDOS predictions, such as more shipment-based transmission in USDOS, using the refined version of USAMM. However, the relative effectiveness of the three control scenarios remained the same despite differences in the underlying livestock movement modeling. We also saw the the outbreak metrics were relatively insensitive to the parameter we introduced to up and down regulate the number of shipments. We added this parameter because with livestock shipments not being tracked there is uncertainty in the total number of shipments occurring. The insensitivity of USDOS predictions to changes in numbers of shipments, suggest that predicted shipment patterns are more important to driving outbreak dynamics. Overall, this suggests that while there is some sensitivity to livestock movement predictions, particularly in the counties that are predicted to have a large proportional difference between the two sets of predictions, the USDOS predictions are robust to uncertainty in the shipment patterns.

Because of the relatively small differences we observed in USDOS in the structural sensitivity analysis, we argue that demographics are an important driver of spatial-temporal spread and geographic patterns of outbreaks. We can also observe this through the sensitivity analysis of county demographic characteristics and their impacts on outbreak metrics. We generally observe larger outbreaks in counties with larger numbers of in-shipments such as in the Midwest or other areas with concentrations of industry infrastructure, which may be further accentuated in counties that additionally have higher density and premises clustering. The super shipper counties observed in the USAMM analysis are also where large outbreaks occur, likely due to their demographic characteristics. We observe smaller outbreaks in counties with a larger number of out-shipments and density, for example counties in the eastern U.S. We also observe smaller outbreaks in counties with a larger number of large premises, but only when those counties also have high density, clustering, and/or out-shipments. One possible interpretation, is that these may be areas that are somewhat industry intensive yet also somewhat isolated from the larger network of the cattle industry. We can use these characteristics to identify regions of interest where it is more likely that large outbreaks could be seeded.

Livestock demographic data in the U.S. is only available in an aggregated form due to data privacy issues. The placement of cattle premises and sizes used in this study were disaggregated and verified by Burdett et al. [18]. To account for the uncertainty in the premises locations we used 10 realizations of the demographic data for every USDOS model scenario [10,18]. If demographic data with less uncertainty should become available in the future, it would improve model predictions by reducing uncertainty in a key driver of outbreak dynamics. However, in the absence of fully accessible data the disaggregated demographic data used in these model simulations gives the most accurate understanding of the spatial structuring of the U.S. cattle population.

From the perspective of understanding FMD in the U.S., it is important to underscore that the results we presented focused on the large outbreaks resulting from our simulations, but that most simulations faded out and did not result in an outbreak at all. We focused on the upper 97.5th percentile of simulated outbreaks in order to understand how a large outbreak would behave in the U.S. should one occur. We also do not incorporate any other species susceptible to FMD in our models, they are exclusively beef and dairy disease models. Including other species may change our predictions, and we are currently exploring the use of USDOS in multispecies outbreaks. Our outcomes may also be affected by our choice to use the same parameter values for both beef and dairy transmission dynamics. Additional caveats to USDOS are discussed in Tsao et al. [10].

Perhaps most importantly, the dominance of demographics versus the importance of these types of model structural assumptions depends on the type of question that needs to be addressed. For some questions, the intensity of the outbreak does matter, and in these cases accurate data, the structural details and exact parameter values also then matter. For example, these details matter when estimating outbreak duration in order to more accurately understand how trade will be impacted. However, simpler simulation assumptions together with accurate demographic data may be enough when questions are more impacted by spatial spread processes and geographic patterns. For example, simpler simulations may be appropriate when trying to understand spatial risk, investigating preferred control options, or planning where to stockpile resources. These simpler approaches are also more computationally efficient, which is an important consideration when timely results are needed for decision-support. Before determining model specifications, it is important to have a clear management objective to tailor your simulations to fit. This will allow for a more useful and efficient process when decisions need to be made quickly [40].

## Figures and Tables

**Table 1:** FMD partial transition parameters.

Parameter	Description	Value
$t_{\sigma }$	Final day on which there are only susceptible or exposed animals	0 days
$t_{S=0}$	Time at which all animals are infectious	4 days
γ	Recovery rate of animals per day	0.44
*r*	Rate of increase of number of infecteds	$r_{0}$ = 0.05, $r_{1}$ = 0.006

**Table 2:** WAIC, and effective sample size values for beef and dairy for models including and excluding covariate data, respectively. As a measure of effective sample size, the number of independent draws from the posterior (IDD) for the parameter with the lowest value for the given model is shown.

Commodity	Data	WAIC	Min. IDD
Beef	Including covariates	323,344	1822.0
	Excluding covariates	345,239	1192.1
Dairy	Including covariates	64,525	6542.9
	Excluding covariates	67,720	7592.4

## Data Availability

At the “U.S. Animal Movement Model and Disease Outbreak Simulation” website (https://webblabb.github.io/usammusdos/index.html), there is a user manual as well as aink to the public GitHub repository which houses the code. The repository can be directly accessed at https://github.com/webblabb/usdos (USDOS c++ code and pre- and post-processing code).

## Supplementary Materials

The following supporting information can be downloaded at: https://www.mdpi.com/article/10.3390/life12101604/s1. The supplementary materials pdf contains supplementary methods and results for USAMM and USDOS [44,45,46,47,48].
