# Supplementary material for: The Importance of Livestock Demography and Infrastructure in Driving Foot and Mouth Disease Dynamics
Source: Life (Basel). 2022 Oct 14;12(10):1604. doi: 10.3390/life12101604 (PMC9605081; doi:10.3390/life12101604)
Supplement: Supplementary file 1 [file life-12-01604-s001.zip › life-1851139-SupplementaryMaterials.pdf]

Supplementary Materials for

The Importance of Livestock Demography and Infrastructure in Driving Foot and Mouth Disease Dynamics

Kendra Gilbertson[a], Peter Brommesson[b], Amanda Minter[c], Clayton Hallman[d], Ryan S. Miller[d], Katie Portacci [d], Stefan Sellman[b], Michael J. Tildesley[c], Colleen T Webb[a], Tom Lindström[b] and Lindsay M. Beck-Johnson[a]

[a]*Department of Biology, Colorado State University, 1878 Campus Delivery, Fort Collins, CO 80523, USA;*
[b]*Department of Physics, Chemistry and Biology, Division of Theoretical Biology, Linköping University, 581 83 Linköping, Sweden*
[c]*Zeeman Institute for Systems Biology and Infectious Disease Epidemiology Research (SBIDER), School of Life Sciences and Mathematics Institute, University of Warwick, Coventry, CV4 7AL, UK;*
[d]*USDA APHIS Veterinary Services, Center for Epidemiology and Animal Health, Fort Collins, CO 80526, USA*

S1. Supplemental Methods

S1.1. Partial Transition Function

In USDOSv2.0 [1], premises are classified as either susceptible, exposed, infectious or immune. When within-herd dynamics are not considered (as in USDOS2.0, Tsao et al. [1]), the rate at which an infectious premises i infects a susceptible premises j is given by,

$$rate_{(i,j)} = ([N^{p_beef}_{(beef,j)}]S_{beef}+[N^{p_dairy}_{(dairy,j)}]S_{dairy})\times([N^{q_beef}_{(beef,i)}]T_{beef}+[N^{q_dairy}_{(dairy,i)}]T_{dairy})\times K(d_{ij}) \quad \text{(S1)}$$

where $N_{(b,i)}$ is the number of individuals of species b on premises i, S_b and T_b are the susceptibility and transmissibility measures for premises of type b and p_b, q_b are power law parameters accounting for a non-linear increase in susceptibility and transmissibility as animal numbers on a premises increase. Infection spreads between premises via the transmission kernel K according to the distance between premises i and j, $d_{(i,j)}$.

For USDOSv2.1, we extend the USDOSv2.0 framework outlined above to incorporate within-herd dynamics such that infectiousness is dependent upon how many animals on the premises are infectious at time t. Therefore, instead of using a fixed premises size $N_{(beef,i)}$ as in USDOSv2.0, we use the number of infectious animals at time t $I(t)_{(beef,i)}$. In this revised model, the rate is now given by,

$$\begin{aligned} rate_{(i,j)} =& ([N^{p_beef}_{(beef,j)}]S_{beef} + [N^{p_dairy}_{(dairy,j)}]S_{dairy}) \times ([I(t)^{q_beef}_{(beef,i)}]T_{beef} \\ &+ [I(t)^{q_dairy}_{(dairy,i)}]T_{dairy}) \times K(d_{ij}) \end{aligned} \quad \text{(S2)}$$

where $I(t)$ is calculated by,

$$I(t) = \mathbb{1}(t < t_{(S=0)})\big(I(0) + \frac{(r + \gamma r(t_{(S=0)} - t))}{(t_{(S=0)}\gamma^2)} - \frac{(r + \gamma r t_{(S=0)})}{(t_{(S=0)}\gamma^2)}\exp(-\gamma t)\big) + \mathbb{1}(t \geq t_{(S=0)})\big(I(0) + \frac{(r\exp(-\gamma t_{(S=0)})) - r - \gamma r t_{(S=0)})}{(t_{(S=0)}\gamma^2)}\exp(-\gamma t)\big) \quad \text{(S3)}$$

and,

$$r = N(r_0 I(t-1) + r_1 t^2) \quad \text{(S4)}$$

where $t_{S=0}$ is the time at which all animals are infectious, γ is the recovery rate of animals per day and r is the rate of increase of number of infectious animals given by the function described in equation S4.

In order to determine the best fit parameters for the partial transition function $I(t)$, we minimized the sum of the squared difference between "data" (mean of 1000 simulations from an FMD within-herd model [2]) and the predicted values from the partial transition function (Table 1).

Figure S1 shows the best fit partial transition function and the mean of the 1000 simulations for different farm sizes (plots) and different numbers of initially exposed animals (colors). The partial transition function provided a good fit to the simulations from the FMD within-herd model [2] across different farm sizes. We did not observe a notable difference in simulations or model fits when we varied the initial number of exposed animals, therefore, we do not include this parameter in the partial transition function.

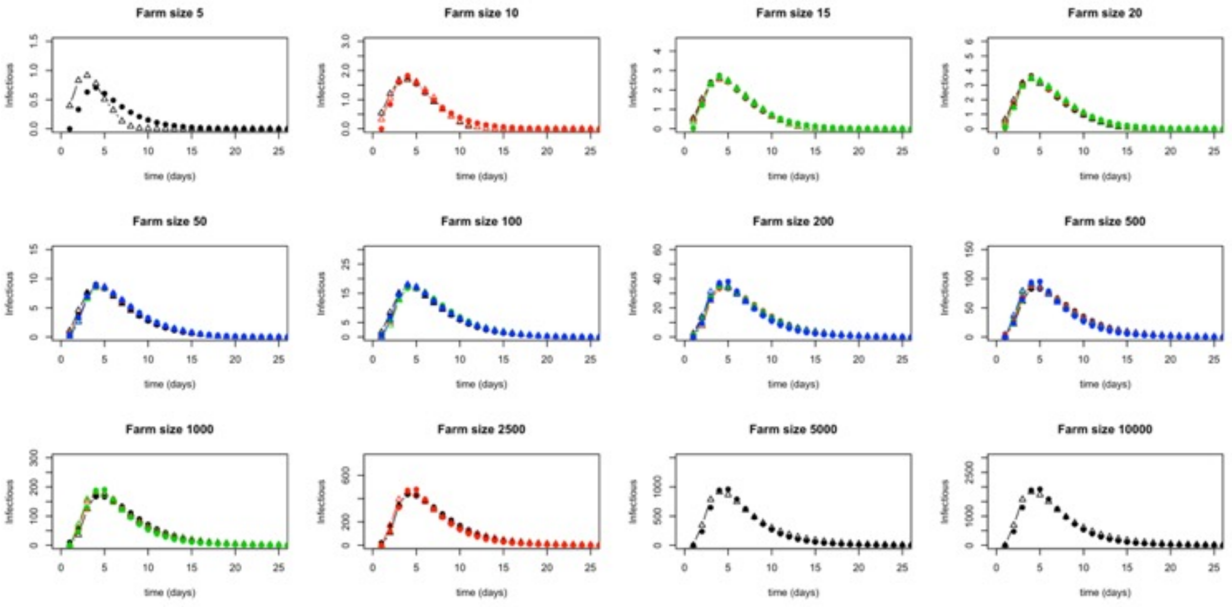

Figure S1: Partial transition function used in USDOSv2.1 curve compared with independent within-herd model (see FMD manuscript on task 2.1 for details) curves for premises ranging from 5 to 10,000 head in size. The circles show the within-herd model simulation results and the triangles show the partial transition function.

The partial transition function describes how many animals are infectious at time t after infection has entered the premises and the latency period has passed. The number of infectious animals is treated as a floating point value rather than an integer, and is referred to as the effective number of infectious animals, I^{eff}:

$$I^{\text{eff}} = f(t, N) = \begin{cases} \frac{r + r\gamma(t_{s0} - t)}{t_{s0}\gamma^2} - \frac{(r + \gamma r t_{s0})}{t_{s0}\gamma^2} e^{-\gamma t}, & \text{if } t < t_{s0} \\ \frac{r e^{\gamma t_{s0}} - r - r\gamma t_{s0}}{t_{s0}\gamma^2} e^{-\gamma t}, & \text{if } t \geq t_{s0} \end{cases}$$

where

$$r = N(r_0 + (t - 1) + r_1 t^2) \tag{S5}$$

and t_{s0}, r_0, r_1 and γ are constants fitted using the FMD within-herd model described in Beck-Johnson et al. [2].
Rewriting r as

$$r = N\rho \tag{S6}$$

and exchanging it into the equations we get

$$I^{\text{eff}} = f(t, N) = \begin{cases} \frac{N\rho + N\rho\gamma(t_{s0} - t)}{t_{s0}\gamma^2} - \frac{(N\rho + \gamma N\rho t_{s0})}{t_{s0}\gamma^2} e^{-\gamma t}, & \text{if } t < t_{s0} \\ \frac{N\rho e^{\gamma t_{s0}} - N\rho - N\rho\gamma t_{s0}}{t_{s0}\gamma^2} e^{-\gamma t}, & \text{if } t \geq t_{s0} \end{cases}$$

N can be broken out of each term and removed to leave f a function of just t multiplied by N:

$$I^{\text{eff}} = f(t, N) = N f(t). \tag{S7}$$

Therefore, the partial transition function becomes a function of time, t, describing the proportion of the premises' population that is infectious after t days such that, regardless of premises size, the proportion will always be the same for each value of t and can be pre-calculated in USDOSv2.1 (Fig. S2). However, simplifying the partial transition function to a function of time t and not herd size, means that the scaling factor to get the same level of transmissibility as in USDOSv2.0 without partial transition is a constant shared by all premises. Looking at R_0 for the infectious premises i, $R_i = \sum_j 1 - e^{-\lambda_{ij}}$. For the case without partial transition (USDOSv2.0, denoted as *noPT* in the equations) the transmission rates are:

$$\lambda_{ij}^{\text{noPT}} = S N_j K(i, j) T N_i P, \tag{S8}$$

where N is the premises sizes, T and S are susceptibility and transmissibility constants, respectively, K is the kernel function, which describes the decay in transmission risk over distance, and P is the infectiousness period (7 days for USDOSv2.0 [1]). Since the effective number of infectious animals is constant in USDOSv2.0, we can say that the effective-infectious-animal-days is a simple multiplication ($N_i * P$).

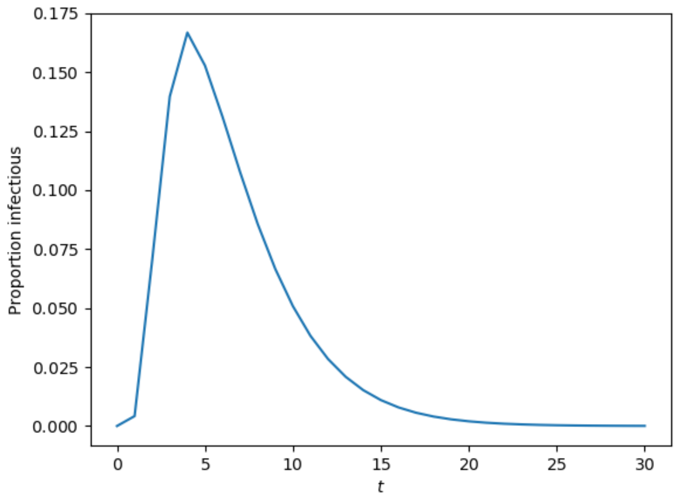

Figure S2: FMD partial transition function $f(t)$.

For the case of USDOSv2.1 with partial transition, the effective-infectious-animal-days is the partial transition function integrated over the period of infectiousness and multiplied by the number of animals on the premises. For one time-step:

$$\lambda_{ij,t}^{\mathrm{PT}} = SN_j K(i,j) T I_{i,t}^{\mathrm{eff.}} \tag{S9}$$

and for the entire period that i is infectious:

$$\lambda_{ij}^{\mathrm{PT}} = SN_j K(i,j) T N_i \int_0^\infty f(t)dt \tag{S10}$$

The area under the curve in Fig. S2 shows the effective-infectious-animal-days over the infectious period. For the case of partial transition in USDOSv2.1 the infectious period is longer than in USDOSv2.0 and the area under the curve (Figure S3) is smaller for the equivalent time frame to the infectious time in USDOSv2.0; this will lead to fewer infections when partial transition is in use. Therefore, we need to scale the area up so it is equivalent to the area of the case without partial transition. We incorporate a scaling factor s in USDOSv2.1 so that:

$$sN_i \int_0^\infty f(t)dt = N_i P, \tag{S11}$$

$$s = \frac{N_i P}{N_i \int_0^\infty f(t)dt}, \tag{S12}$$

$$s = \frac{P}{\int_0^\infty f(t)dt}, \tag{S13}$$

Since the integral (given a set of within-herd model parameters) and the infectious period, P, are constants, the scaling factor is also a constant.

The limits that are used in the integration will have an effect on the scaling factor. If the limits of integration are 0 and infinity (∞), which is the most exact case, it leads to premises that never stop being infectious in the absence of interventions. Without the partial transition of states, we implement a set period of infectiousness after which the premises becomes immune, regardless of intervention or absence thereof. While we could allow the partial transition function to be evaluated to infinity, it is not biologically realistic and it would be computationally costly. We therefore, assign a maximum infectious period, such that premises are infectious at a level determined by the partial transition function until the maximum infectious period is reached after which they become immune. To ensure that we capture the biologically relevant aspects of the partial transition function, the maximum infectious period is selected from the tail of the distribution, 15 or more days (Figure S2).

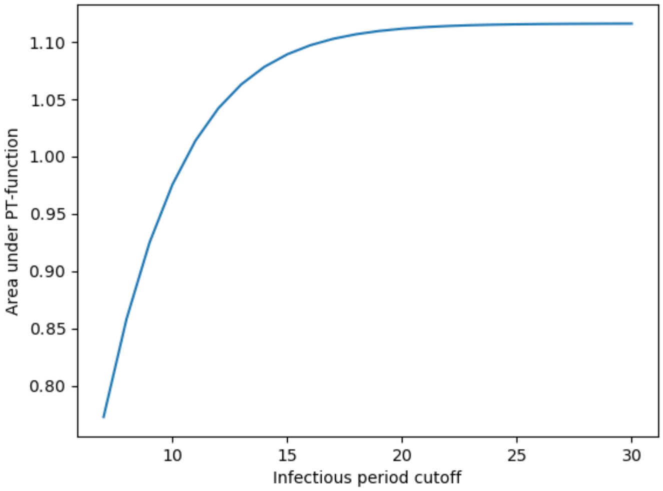

Figure S3: Area under the FMD partial transition function increases and then plateaus with increasing infectious period cutoff.

The selection of an appropriate USDOSv2.1 default value for maximum infectious period requires an exploration of how changing the cut-off affects the results. We therefore ran simulations without control for three different cut-off times. Based on the area under the curve analysis, we selected cut-off times of 15, 20 and 30 days, respectively. The scaling factor value for each of the three maximum infectious period values are given in Table S1.

Table S1: Partial Transition Function Cut-off and Scaling Parameters

**Parameter**	**Value**	**Scaling Factor** s
Maximum infectious period	15 days	6.49195
	20 days	6.30852
	30 days	6.27237

The three maximum infectious period scenarios were set up with the same parameters, settings, and input files as were run for USDOSv2.0 no control simulations (Table S2) [1]. Simulations were seeded in each of the 3049 counties in the contiguous U.S. 10 times for each of the 10 FLAPS realizations, which leads to each county being seeded 100 times. Each time a county is seeded a farm is selected at random to be the source of the outbreak.

Table S2: USDOSv2 Transmission Parameters

**Parameter**	**Default Value**	**Range**	**Reference**
Cattle transmission rate (a_c)	10.252	3.6–100	[1]
Cattle susceptibility (b_c)	1	N/A	[1]
Normalising constant (k_1)	$1.46e^{-08}$	$4.07e^{-10}$–$3.91e^{-08}$	[1]
Scale parameter for spatial kernel (k_2)	1686.16	1686.16–5414.72	[1]
Shape parameter for spatial kernel (k_3)	2.267	2.022–3.006	[1]
Latency period	5 days	3–13 days	[1]

S1.2. USAMMv2.0 Model Definition

In USAMMv2.1, we build on the earlier USAMMv2.0 movement model of [4] to improve the previous statistical model for the the probability of observing a set of interstate cattle shipments given by the 2009 ICVI data. In this section we describe this previous model, and in the next section outline the changes that were made to it in the present work. Briefly, it was assumed in USAMMv2.0 that within each state $u \in \mathbf{U}$, where $\mathbf{U}$ is the set of states in the contiguous U.S., shipments arose from each single premises as a Poisson process with the premises-level shipment rate λ^*_u. Given this assumption, the collective rate with which shipments arose from each origin county $\omega \in \mathbf{V}_u$ was simply the number of premises in the county multiplied by $\lambda^*_{u(\omega)}$. Here $\mathbf{V}_u$ is the set of all counties in u, and, in order to minimize the use of subscripts, we adopt the notation $u(v)$ to denote the state in which an arbitrary county v is situated (i.e. in this case $u(\omega)$ implies the state of the origin county ω). The

shipments that arose from ω were then distributed among all possible destination counties $\delta \in \mathbf{V}$, where $\mathbf{V}$ is the set of all counties of all states in $\mathbf{U}$. This last step was achieved by assigning each δ a weight based on an attraction parameter of the destination state, $\nu_{u(\delta)}$, as well as a function of the distance between ω and δ and two origin state-level parameters, $\kappa_{u(\omega)}$ and $\pi_{u(\omega)}$. Given these weights, the probability that a shipment originating in ω would have δ as destination could be determined.

With the above definitions, and denoting the complete set of shipments occurred as $\mathbf{Y}$, the probability of the number of shipments $Y_{\omega,\delta,\tau} = |\mathbf{Y}_{\omega,\delta,\tau}|$ in each subset $\mathbf{Y}_{\omega,\delta,\tau} \in \mathbf{Y}$ of shipments moving from origin county ω to destination county δ on day τ (1 January being $\tau = 1$) was expressed as

$$\mathrm{P}\left(Y_{\omega,\delta,\tau}\right) = \mathrm{P}\left(Y_{\omega,\tau}|\boldsymbol{\theta}_{Q(\tau)}\right)\mathrm{P}\left(\delta|\omega,\boldsymbol{\theta}_{Q(\tau)}\right). \tag{S14}$$

This is the joint probability of $Y_{\omega,\tau}$ shipments leaving county ω, and that δ is the destination county conditional on ω being the origin. USAMMv2.0 included a component of seasonality with parameters being defined for each quarter of the year, and the full set of model parameters for a quarter is here denoted by $\boldsymbol{\theta}_{Q(\tau)}$, where $Q(\tau)$ is the quarter into which the day τ falls.

The number of shipments to originate from a county ω each day was assumed to be Poisson distributed, with a daily shipment rate $\lambda_{\omega,Q(\tau)}$,

$$Y_{\omega,\tau} \sim \mathrm{Pois}\left(\lambda_{\omega,Q(\tau)}\right), \tag{S15}$$

and the first factor of equation (S14) is consequently given by the probability mass function of the Poisson distribution:

$$\mathrm{P}\left(Y_{\omega,\tau}|\boldsymbol{\theta}_{Q(\tau)}\right) = \frac{\lambda_{\omega,Q(\tau)}^{Y_{\omega,\tau}} e^{-\lambda_{\omega,Q(\tau)}}}{Y_{\omega,\tau}!}. \tag{S16}$$

The daily shipment rate of the county ω was given by scaling a state- and quarter-specific premises-level shipment rate by the total number of premises in the origin county so that

$$\lambda_{\omega,Q(\tau)} = w_\omega \lambda^*_{u(\omega),Q(\tau)}. \tag{S17}$$

Here, $\lambda^*_{u,Q(\tau)}$ is the state- and quarter-specific rate with which shipments originate from each single premises within the state of u and was estimated as a parameter in the model. To obtain the county-level shipment rate, $\lambda^*_{u,Q(\tau)}$ was simply multiplied by the number of premises in the county, i.e. denoting the set of all premises in a county v as $\mathbf{P}_v$, w_ω was given by

$$w_v = \sum_{i \in \mathbf{P}_v} 1. \tag{S18}$$

The second factor of equation (S14) describes how likely one of the $Y_{\omega,\tau}$ shipments that originated from ω was to end up in each of all the possible destination counties. This process was controlled by two separate mechanisms. First, each state u was associated with a parameter, $\nu_{u,Q}$, controlling the propensity for a single premises in the state to attract

shipments in quarter Q. When scaled up by the number of premises in a county, this gave a total measure of the entire county's capacity to attract shipments based on the size of its population of premises. Second, each state was associated with a monotonically decreasing distance kernel function, H, which gave a distance dependence component to the county–to–county shipment probability. In [4], three different kernel functions were implemented and compared, of which the functional form

$$H(d,s,m) = \frac{1}{1+\left(\frac{d}{s}\right)^m}, \tag{S19}$$

was preferred in model selection. The two state-specific parameters $s_{u,Q}$ and $m_{u,Q}$ determines the kernel's scale and shape. This spatial kernel (with the same functional form as the kernel used for local transmission, eq. 2), is plateau-shaped at short distances and has a fat tail describing the probability of long distance shipments.

To make the kernel parameters easier to interpret, the distance kernel function was reparameterized to be expressed with parameters $\kappa_{u,Q}$ and $\pi_{u,Q}$. The scale measure $\kappa_{u,Q}$ was defined as the distance (in kilometers) to where the value of the kernel had dropped to 50% of its value at distance zero, i.e. the distance where a premises is half as likely to be the destination as an immediate neighboring premises, all other things equal. The scale parameter $\pi_{u,Q}$, was defined as the ratio between $\kappa_{u,Q}$ and the distance where the kernel value is 0.05% of its value at zero. The choice of 0.05% was somewhat arbitrary but corresponds to a substantially lower kernel value compared to the value at distance $\kappa_{u,Q}$. Expressing the kernel with parameters that offer an intuitive sense of the kernel behavior promotes prior elicitation and interpretation of results. Omitting state and quarter indices for brevity, parameters s and m in equation (S19) are calculated from κ and π as

$$s = \frac{\kappa}{\exp\left[\frac{\ln\left(\frac{1-x_1}{x_1}\right)}{m}\right]} \tag{S20}$$

and

$$m = \frac{\ln\left(\frac{1-x_1}{x_1}\right) - \ln\left(\frac{1-x_2}{x_2}\right)}{\ln \pi} \tag{S21}$$

To consider strictly monotonically decreasing kernels and positive shipment distances only, the parameters were restricted to $\kappa_{u,Q} \in (0,\infty)$ and $\pi_{u,Q} \in (1,\infty)$. Defining $\pi_{u,Q} > 1$ ensured that no kernels had a reverse distance dependence, i.e. distance dependence where the probability of shipment increases with distance, which was deemed unlikely for the system.

Given an origin county ω and the above two mechanisms, a weight was given to each destination county δ, describing how likely it was to receive a shipment from ω in relation to all other counties,

$$W_{\omega,\delta,Q(\tau)} = w_\delta \nu_{u(\delta),Q(\tau)} H\left(\kappa_{u(\omega),Q(\tau)}, \pi_{u(\omega),Q(\tau)}, d_{\omega,\delta}\right). \tag{S22}$$

Here, w_δ was defined as the number of premises in δ, (equation S18), and the product given by $w_\delta \nu_{u(\delta),Q(\tau)}$ was the total county-level attraction contributed by all the premises in δ. The inter-county distance, $d_{\omega,\delta}$, was given by the Euclidean distance in kilometers between the two counties' centroids, and scaled the weight via the distance kernel function of the state in which the origin county is located. By normalizing $W_{\omega,\delta,Q(\tau)}$ over all possible destination counties, an expression for the probability that any shipment originating in ω goes to δ (i.e. the second half of equation S14) was obtained,

$$\mathrm{P}\left(\delta|\omega, \boldsymbol{\theta}_{Q(\tau)}\right) = \frac{W_{\omega,\delta,Q(\tau)}}{\sum_{i \in \mathbf{V}} W_{\omega,i,Q(\tau)}}. \tag{S23}$$

S1.3. USAMMv2.1

In the above model definition of USAMMv2.0, each premises is given equal weight through the equation (S18), and the variables w_ω and w_δ are simply the number of premises in the respective county. Analyses of other systems have however revealed that both the number of incoming and outgoing shipments vary with the type and herd size of the premises [5]. In USAMMv2.1, we therefore relaxed the assumption that all premises send and receive the same number of shipments and model the contribution of each individual premises i as a function of its herd size, h_i (i.e. number of animals). We assumed that this relationship between herd size and probability to receive or send shipments is dependent on the type of premises and its size. We include three such types: farms (F), feedlots (E), and markets (M). For each county v, we defined three corresponding subsets of premises: $\mathbf{P}_v^{\mathrm{F}}$, $\mathbf{P}_v^{\mathrm{E}}$, and $\mathbf{P}_v^{\mathrm{M}}$, each consisting of the sub-population of premises of the three types within the county. Further, we assume that the relationship between h_i and the probability to receive shipments is independent from the relationship between h_i and the probability of sending shipments. Therefore we redefine w_v to be two separate variables denoted $\check{w}_v$ when v is acting as the receiving county, and $\hat{w}_v$ when v is the origin county. Last, we assume that the relationships between h_i and shipment probability can be nonlinear and model them in the form of a set of power laws with parameters specific to type (g), quarter, and direction:

$$\check{w}_{v,\tau} = \sum_{g \in \mathbf{G}} \sum_{i \in \mathbf{P}_v^g} \left[\check{\psi}_{g,Q(\tau)} \left(\frac{h_i}{\bar{h}_g} \right)^{\check{\phi}_{g,Q(\tau)}} \right] \tag{S24}$$

for receiving counties, and

$$\hat{w}_{v,\tau} = \sum_{g \in \mathbf{G}} \sum_{i \in \mathbf{P}_v^g} \left[\hat{\psi}_{g,Q(\tau)} \left(\frac{h_i}{\bar{h}_g} \right)^{\hat{\phi}_{g,Q(\tau)}} \right] \tag{S25}$$

for sending counties. Here parameters $\check{\psi}_{g,Q}$ and $\hat{\psi}_{g,Q}$ control the magnitude of the relationship while $\check{\phi}_{g,Q}$ and $\hat{\phi}_{g,Q}$ govern how the effect scales with herd size (with the accent indicating directionality). To make parameters easier to interpret and comparable across types, the herd sizes of the premises of each type g are normalized over the average size of all premises

of that type across the entire U.S. (i.e. $\bar{h}_g = \sum_{i \in \mathbf{P}_g} h_i \cdot |\mathbf{P}_g|^{-1}$). In effect this means that rather than evaluating the effect of a premises of a certain size, we evaluate the effect of a premises size in relation to the effect of a premises of average size. The normalization means that for a premises of average size (for its specific type) the quantity in brackets in equations (S24) and (S25) will evaluate to 1.0, and the parameters $\check{\psi}_{g,Q}$ and $\hat{\psi}_{g,Q}$ can consequently be interpreted directly as the effect of a premises of average size. Further, for the farm type we set $\check{\psi}_{\mathrm{F},Q} = 1.0$ and $\hat{\psi}_{\mathrm{F},Q} = 1.0$ in order to express the effect of feedlots and markets in relation to farms (i.e. an average sized farm will always have the weight 1.0). For premises of types farm and feedlot, h_i reflect the actual herd size, but, for markets, we use the total yearly volume (measured in head) as herd size.

In addition to the differentiation between premises types and premises of different size, we introduce a county-level effect in the form of county-level livestock industry covariates. We therefore introduce the set of covariates $\mathbf{C}$, and for each covariate $c \in \mathbf{C}$ we define the vector $\boldsymbol{\zeta}_c$ with one element for each county. Each separate vector was standardized to have a mean of zero and standard deviation of 0.5. The covariates included in $\mathbf{C}$ were operations with sales (OS), total sales (in head, TS) and slaughter connectivity (SC). Slaughter connectivity is a measure of the available slaughter capacity in the vicinity of the county v and was calculated as the distance-weighted sum of annual slaughter volume in all other counties,

$$\zeta_{\mathrm{SC},v} = \sum_{\substack{x \in \mathbf{V} \\ x \neq v}} l_x e^{-d_{vx}/125}, \tag{S26}$$

where l_x is the slaughter volume in head of county x, $\mathbf{V}$ is the set of all counties, and d_{vx} is the distance between the centroids of v and x. The distance is normalized over an estimated average slaughter shipment distance in the U.S. of 125 km [3]. The effect of these covariates on the weighing of each county v was given by

$$\check{z}_{v,\tau} = \exp\left[\sum_{c \in \mathbf{C}} \zeta_{c,v} \check{\eta}_{c,Q(\tau)}\right] \tag{S27}$$

for the effect when the county v is acting as receiver of shipments, and

$$\hat{z}_{v,\tau} = \exp\left[\sum_{c \in \mathbf{C}} \zeta_{c,v} \hat{\eta}_{c,Q(\tau)}\right] \tag{S28}$$

when v is acting as the sender. Here, $\check{\eta}_{c,v,Q(\tau)}$ and $\hat{\eta}_{c,v,Q(\tau)}$ are model parameters that control the effect of each covariate c in quarter Q. Once again directionality is indicated by the accent above the parameter. The normalization of the individual covariate vectors means that for an average county (i.e. a county for which all covariates equal the national mean, 0.0), the covariates have no effect ($z_v = 1.0$). This is also true if all parameters weighting the covariates ($\check{\eta}_{c,Q}$ and $\hat{\eta}_{c,Q}$ are zero for all values of c).

We incorporate the additions of these premises- and county-level weights into the definition of USAMMv2.0 by reformulating equations (S17) and (S22). For the shipment rate of

the origin county ω, the weight contributed by the premises originally given by the number of premises in the county (w_ω, equation S18) is exchanged for the weight scaled according to premises type and herd size ($\hat{w}_\omega$, equation S25). Additionally, the weight contributed by the premises population of the origin county is also scaled with the county-level effect of industry covariates ($\hat{z}_\omega$, equation S28):

$$\lambda_{\omega,Q(\tau)} = \hat{z}_{\omega,\tau}\hat{w}_{\omega,\tau}\lambda^*_{u(\omega),Q(\tau)}. \tag{S29}$$

While in the previous version of the model, $\lambda^*_{u,Q}$, reflected the shipment rate of any single premises within state u, with the changes to USAMMv2.1 it is better interpreted as the rate of shipment of an average sized farm in an average county with regard to industry covariates.

For county destination weight (equation S22), the premises count of the destination county δ was similarly exchanged for premises- and covariate weights to get

$$W_{\omega,\delta,Q(\tau)} = \check{z}_{\delta,\tau}\left(\check{w}_{\delta,\tau} - L_{\delta|\omega,\tau}\right)\nu_{u(\delta),Q(\tau)}H\left(\kappa_{u(\omega),Q(\tau)}, \pi_{u(\omega),Q(\tau)}, d_{\omega,\delta}\right). \tag{S30}$$

The function of the term $L_{\delta|\omega,\tau}$ is to account for the reasonable assumption that a premises cannot send shipments to itself and was defined as

$$L_{\delta|\omega,\tau} = \begin{cases} \dfrac{\displaystyle\sum_{g\in\mathbf{G}}\sum_{i\in\mathbf{P}^g_\delta}\left[\check{\psi}_{g,Q(\tau)}\left(\frac{h_i}{\bar{h}_g}\right)^{\check{\phi}_{g,Q(\tau)}}\hat{\psi}_{g,Q(\tau)}\left(\frac{h_i}{\bar{h}_g}\right)^{\hat{\phi}_{g,Q(\tau)}}\right]}{\displaystyle\sum_{g\in\mathbf{G}}\sum_{i\in\mathbf{P}^g_\delta}\left[\hat{\psi}_{g,Q(\tau)}\left(\frac{h_i}{\bar{h}_g}\right)^{\hat{\phi}_{g,Q(\tau)}}\right]} & \delta = \omega, \\ 0 & \text{otherwise.} \end{cases} \tag{S31}$$

That is, $L_{\delta|\omega,\tau}$ corresponds to the sum of the weights related to being the destination of a shipment, weighted by their origin weights.

The state level attraction parameter $\nu_{u,Q}$ and the distance kernel function H and associated parameters $\kappa_{u,Q}$ and $\pi_{u,Q}$ remained unchanged from the USAMMv2.0 definition. In order to make the model identifiable, the attraction parameter for the state of Iowa was set to one ($\nu_{\text{Iowa},Q} = 1.0$). The choice of Iowa was somewhat arbitrary, but its large number of shipments promotes high precision of $\nu_{\text{Iowa},Q} = 1.0$, which facilitates computational efficiency in estimation of the other states' parameters, which are contrasted against this reference state.

Despite the new additions, the likelihood function of USAMMv2.1 is equivalent to that defined for USAMMv2.0 and is obtained by injecting the variables defined in equations (S29) and (S30) into equations (S16) and (S23). Via the weights $\check{w}_{\delta,\tau}$, $\hat{w}_{\omega,\tau}$, $\check{z}_{\delta,\tau}$ and $\hat{z}_{\omega,\tau}$ the new model allows for the inclusion of county and premises level heterogeneities. However it is also possible for the model to disregard one or both of them for certain parameter values. The latter is equivalent to the base model in [4] which can be viewed as a special case of USAMMv2.1 where each county has an effect of industry covariates equal to one (i.e. no effect of covariates), and where every premises has equal weight regardless of size and type.

S1.4. USAMM Likelihood

We followed [4] and modeled through eq. S14 the total probability of observing $Y_{\omega,\delta,\tau} = |\mathbf{Y}_{\omega,\delta,\tau}|$ number of interstate shipments from county ω on day τ to all other counties $\delta \in \bar{\mathbf{V}}_{\mathbf{u}(\omega)}$, where $\bar{\mathbf{V}}_{\mathbf{u}(\omega)} = \mathbf{V} \backslash \mathbf{V}_{u(\omega)}$ (i.e. all counties not in the origin state) as a product of the individual probabilities. Here, we exclude within state shipments since they are absent in the ICVI data. This probability is equivalent to the probability of observing the shipments in the set $\mathbf{Y}_{\omega,\tau} = \bigcup_{\delta \in \bar{\mathbf{V}}_{\mathbf{u}(\omega)}} \mathbf{Y}_{\omega,\delta,\tau}$.

For notational convenience, we introduce the following definitions:

$$\boldsymbol{\Theta}_{u,Q} =(\kappa_{u,Q}, \pi_{u,Q}), \tag{S32}$$
$$\boldsymbol{\Theta}_{u} =(\boldsymbol{\Theta}_{u,1}, \ldots, \boldsymbol{\Theta}_{u,4}), \tag{S33}$$
$$\boldsymbol{\Theta} =(\boldsymbol{\Theta}_{1,1}, \ldots, \boldsymbol{\Theta}_{|\mathbf{U}|,4}), \tag{S34}$$
$$\boldsymbol{\lambda}_{Q} =(\lambda^*_{1,Q}, \ldots, \lambda^*_{|\mathbf{U}|,Q}), \tag{S35}$$
$$\boldsymbol{\nu}_{Q} =(\nu_{1,Q}, \ldots, \nu_{|\mathbf{U}|,Q}), \tag{S36}$$
$$\boldsymbol{\psi}_{Q} =(\check{\psi}_{\mathrm{F},Q}, \check{\psi}_{\mathrm{E},Q}, \check{\psi}_{\mathrm{M},Q}, \hat{\psi}_{\mathrm{F},Q}, \hat{\psi}_{\mathrm{E},Q}, \hat{\psi}_{\mathrm{M},Q}), \tag{S37}$$
$$\boldsymbol{\phi}_{Q} =(\check{\phi}_{\mathrm{F},Q}, \check{\phi}_{\mathrm{E},Q}, \check{\phi}_{\mathrm{M},Q}, \hat{\phi}_{\mathrm{F},Q}, \hat{\phi}_{\mathrm{E},Q}, \hat{\phi}_{\mathrm{M},Q}), \tag{S38}$$
$$\boldsymbol{\eta}_{Q} =(\check{\eta}_{1,Q}, \ldots, \check{\eta}_{|\mathbf{C}|,Q}, \hat{\eta}_{1,Q}, \ldots, \hat{\eta}_{|\mathbf{C}|,Q}). \tag{S39}$$

Since $\lambda_{\omega,Q(\tau)}$ described the rate with which shipments originating from ω arose, it was necessary to account for that the ICVI data was not a 100% sample. This was included in the variable ϵ_u, which reflected this. Since the ICVI data set consisted of a 10% sample of all outgoing ICVI shipments from every state apart from New Jersey, $\epsilon_u = 0.0$ for this state and $\epsilon_u = 0.1$ for all other states in the contiguous U.S. We therefore introduce $\tilde{\lambda}_{u(\omega),Q} = \epsilon_{u(\omega)}\lambda_{(\omega),Q}$. We also let $q_{\omega,Q} = \sum_{\delta \in \bar{\mathbf{V}}_{\mathbf{u}(\omega)}} p_{(\delta|\omega),Q}$, where $p_{(\delta|\omega),Q}$ is given by equation (S23), i.e. the probability of a shipment from county ω leaving the state. This allows us to write the sought probability as

$$\begin{aligned}
&P_{\omega,\tau}\left(\mathbf{Y}_{\omega,\tau}|\boldsymbol{\Theta}_{u(\omega),Q(\tau)}, \lambda^*_{u(\omega),Q(\tau)}, \boldsymbol{\nu}_{Q(\tau)}, \boldsymbol{\psi}_{Q(\tau)}, \boldsymbol{\phi}_{(Q(\tau)}, \boldsymbol{\eta}_{Q(\tau)}\right) = \\
&\prod_{\delta \in \bar{\mathbf{V}}_{\mathbf{u}(\omega)}} \text{Poisson}\left(Y_{\omega,\delta,\tau}\,\middle|\,\lambda^*_{\omega,Q(\tau)} p_{(\delta|\omega),Q(\tau)}\right) = \\
&\text{Poisson}\left(|\mathbf{Y}_{\omega,\tau}|\,\middle|\,\lambda^*_{u(\omega),Q(\tau)} q_{\omega,Q(\tau)}\right) \text{MN}\left(\begin{pmatrix} |\mathbf{Y}_{\omega,\tau,1}| \\ \vdots \\ |\mathbf{Y}_{\omega,\tau,|\bar{\mathbf{V}}_{\mathbf{u}(\omega)}|}| \end{pmatrix} \middle| |\mathbf{Y}_{\omega,\tau}|, \begin{pmatrix} \frac{p_{(1|\omega),Q(\tau)}}{q_{\omega,Q(\tau)}} \\ \vdots \\ \frac{p_{\left(|\bar{\mathbf{V}}_{\mathbf{u}(\omega)}|\middle|\omega\right),Q(\tau)}}{q_{\omega,Q(\tau)}} \end{pmatrix}\right),
\end{aligned} \tag{S40}$$

where MN denotes the multinomial distribution.

Again following [4], we modeled the probability considering all origin counties in state S and all days in the considered quarter, $\boldsymbol{\tau}(Q)$, of year 2009 in the same way as with $P_{\omega,\tau}\left(\mathbf{Y}_{\omega,\tau}|\boldsymbol{\Theta}_{u(\omega),Q(\tau)}, \lambda^*_{u(\omega),Q(\tau)}, \boldsymbol{\nu}_{Q(\tau)}, \boldsymbol{\psi}_{Q(\tau)}, \boldsymbol{\phi}_{(Q(\tau)}, \boldsymbol{\eta}_{Q(\tau)}\right)$ in eq. S40. That is, we modeled the probability of observing the set of transports $\mathbf{Y}_{S,\boldsymbol{\tau}(Q)} = \bigcup_{\omega \in \mathbf{V}_S} \bigcup_{\tau \in \boldsymbol{\tau}(Q)} \mathbf{Y}_{\omega,\tau}$ as a product of

the individual probabilities expressed in eq. S40.

With $r_{S,Q(\tau)} = \sum_{\omega \in \mathbf{V}_S} \left(\hat{w}_{\omega,\tau} \hat{z}_{\omega,\tau} \sum_{\tau \in \tau(Q)} q_{\omega,Q(\tau)} \right)$, this is written as

$$\begin{gathered} P_{S,Q}\left(\mathbf{Y}_{S,\boldsymbol{\tau}(\boldsymbol{Q})} \left| \boldsymbol{\Theta}_{S,Q(\tau)}, \lambda^*_{S,Q(\tau)}, \boldsymbol{\nu}_{Q(\tau)}, \boldsymbol{\psi}_{Q(\tau)}, \boldsymbol{\phi}_{Q(\tau)}, \boldsymbol{\eta}_{Q(\tau)} \right.\right) = \\ \prod_{\omega \in \mathbf{V}_S} \prod_{\tau \in \boldsymbol{\tau}(Q)} P_{\omega,\tau}\left(\mathbf{Y}_{\omega,\tau} \left| \boldsymbol{\Theta}_{u(\omega),Q(\tau)}, \lambda^*_{u(\omega),Q(\tau)}, \boldsymbol{\nu}_{Q(\tau)}, \boldsymbol{\psi}_{Q(\tau)}, \boldsymbol{\phi}_{Q(\tau)}, \boldsymbol{\eta}_{Q(\tau)} \right.\right) = \\ \text{Poisson}\left(|\mathbf{Y}_{S,\boldsymbol{\tau}(Q)}| \left| \tilde{\lambda}_{S,Q} |\boldsymbol{\tau}(Q)| r_{S,Q(\tau)} \right.\right) \text{MN}\left(\left(\begin{array}{c} |\mathbf{Y}_{1,1,1}| \\ \vdots \\ |\mathbf{Y}_{|\mathbf{V}_S|,|\bar{\mathbf{V}}_S|,|\boldsymbol{\tau}(Q)|}| \end{array} \right) \left| |\mathbf{Y}_{S,\boldsymbol{\tau}(Q)}|, \left(\begin{array}{c} \frac{p_{1,1,1}}{r_{S,Q} \times |\boldsymbol{\tau}(Q)|} \\ \vdots \\ \frac{p_{(|\mathbf{V}_S||\bar{\mathbf{V}}_S|),Q}}{r_{S,Q} \times |\boldsymbol{\tau}(Q)|} \end{array} \right) \right.\right). \end{gathered} \tag{S41}$$

The full likelihood considering all shipments $\mathbf{Y}$ is obtained by multiplying the individual state level probabilities in eq. S41. Thus, it is written as

$$\begin{gathered} L_{\mathbf{Y}}\left(\mathbf{Y} | \boldsymbol{\Theta}, \boldsymbol{\lambda}, \boldsymbol{\nu}, \boldsymbol{\psi}, \boldsymbol{\phi}, \boldsymbol{\eta}\right) = \\ \prod_{Q \in \mathbf{Q}} \prod_{S \in \mathbf{U}} P_{S,Q}\left(\mathbf{Y}_{S,\boldsymbol{\tau}(Q)} \left| \boldsymbol{\Theta}_{S,Q}, \lambda^*_{S,Q}, \boldsymbol{\nu}_Q, \boldsymbol{\psi}_Q, \boldsymbol{\phi}_Q, \boldsymbol{\eta}_Q \right.\right) \end{gathered} \tag{S42}$$

S1.5. USAMM Hierarchical Bayesian Model

A benefit of the Bayesian paradigm is that it provides a joint probability distribution of all random variables. This parameter uncertainty is straightforward to carry forward to the predictive stage by sampling repeatedly from the posterior distribution. Further, we make use of hierarchical modelling, which allows for so called *borrowing strength*. In this case, it means that estimation of state-level parameters, in particular for states with weak data, is improved through the assumption that all state level parameters come from some U.S. distribution, which is described by parameters also estimated in the model.

We chose to model $\kappa_{S,Q} \in \boldsymbol{\kappa}, \pi_{S,Q} \in \boldsymbol{\pi}$ and $\lambda^*_{S,Q} \in \boldsymbol{\lambda}$, where bold symbols denote sets including corresponding parameters for all states and quarters, with hierarchical structure. Here, we implement

$$\begin{aligned} \kappa_{S,Q} &\sim \text{Log-normal}\left(m_{\boldsymbol{\kappa}}, k_{\boldsymbol{\kappa}}\right) \\ \pi_{S,Q} &\sim \text{Log-normal}_{m-1}\left(m_{\boldsymbol{\pi}}, k_{\boldsymbol{\pi}}\right) \\ \lambda^*_{S,Q} &\sim \text{Gamma}\left(m_{\boldsymbol{\lambda}}, k_{\boldsymbol{\lambda}}\right) \end{aligned} \tag{S43}$$

as prior distributions. In S43, m and k represent mean and coefficient of variation for the different parameters (specified as subscripts) and $m-1$ indicates that we use the distribution shifted one unit to account for the assumption of $\pi_{S,Q} > 1$. The log-normal distribution is typically parameterized by the mean and standard deviation of the logarithm of the variable, and we denote these parameters as μ and σ, respectively. The relationships to the prior parameters for $\kappa_{S,Q}$ in S43 are $m_{\boldsymbol{\kappa}} = e^{\mu - \frac{\sigma^2}{2}}$ and $k_{\boldsymbol{\kappa}} = (e^{\sigma^2} - 1)^{\frac{1}{2}}$. Similarly, for $\pi_{S,Q}$ (S43) the relationships are expressed as $m_{\boldsymbol{\pi}} = e^{\mu - \frac{\sigma^2}{2}} + 1$ and $k_{\boldsymbol{\pi}} = (e^{\sigma^2} - 1)^{\frac{1}{2}}$, with the difference in that $m_{\boldsymbol{\pi}}$ adjusts for the distribution being shifted. Further, the relationships to shape (α) and rate (β) in a standard parameterization of the gamma distribution are $m = \alpha\beta^{-1}$ and $k = \alpha^{-\frac{1}{2}}$. In S43, m and k are hyperpriors that we estimate in the hierarchical structure of

the Bayesian model. As such, they require specification of hyperprior distributions, which is elaborated on in S1.6.

Using the notation Φ_l and Ψ_l for prior and hyperprior, respectively, for parameter l, we specify the full Bayesian model as

$$\begin{aligned}
&P_{\mathbf{Y}}\left(\boldsymbol{\Theta}, \boldsymbol{\lambda}, \boldsymbol{\nu}, \boldsymbol{\psi}, \boldsymbol{\phi}, \boldsymbol{\eta}, m_{\boldsymbol{\kappa}}, k_{\boldsymbol{\kappa}}, m_{\boldsymbol{\pi}}, k_{\boldsymbol{\pi}}, m_{\boldsymbol{\lambda}}, k_{\boldsymbol{\lambda}} | \mathbf{Y}\right) \propto \\
&L_{\mathbf{Y}} \prod_{Q \in \mathbf{Q}} \prod_{S \in \mathbf{U}} \left[\Phi_{\boldsymbol{\kappa}}\left(\kappa_{S,Q} | m_{\boldsymbol{\kappa}}, k_{\boldsymbol{\kappa}}\right) \Phi_{\boldsymbol{\pi}}(\pi_{S,Q} | m_{\boldsymbol{\pi}}, k_{\boldsymbol{\pi}}) \Phi_{\boldsymbol{\lambda}}(\lambda^*_{S,Q} | m_{\boldsymbol{\lambda}}, k_{\boldsymbol{\lambda}}) \Phi_{\boldsymbol{\nu}}(\nu_{S,Q})\right] \cdot \\
&\prod_{Q \in \mathbf{Q}} \left(\prod_{g \in \{\mathrm{E,M}\}} \Phi_{\boldsymbol{\psi}}(\check{\psi}_{\mathrm{g},Q}) \Phi_{\boldsymbol{\psi}}(\hat{\psi}_{\mathrm{g},Q}) \prod_{g \in \{\mathrm{F,E,M}\}} \Phi_{\boldsymbol{\phi}}(\check{\phi}_{g,Q}) \Phi_{\boldsymbol{\phi}}(\hat{\phi}_{g,Q}) \Phi_{\boldsymbol{\eta}}(\check{\eta}_{c,Q}) \Phi_{\boldsymbol{\eta}}(\hat{\eta}_{c,Q}) \right) \cdot \\
&\Psi^{(\boldsymbol{\kappa})}_m(m_{\boldsymbol{\kappa}}) \Psi^{(\boldsymbol{\kappa})}_k(k_{\boldsymbol{\kappa}}) \Psi^{(\boldsymbol{\pi})}_m(m_{\boldsymbol{\pi}}) \Psi^{(\boldsymbol{\pi})}_k(k_{\boldsymbol{\pi}}) \Psi^{(\boldsymbol{\lambda})}_m(m_{\boldsymbol{\lambda}}) \Psi^{(\boldsymbol{\lambda})}_k(k_{\boldsymbol{\lambda}}) = \\
&\prod_{Q \in \mathbf{Q}} \prod_{S \in \mathbf{U}} \left[P_S(\mathbf{T}_{S,Q} | \boldsymbol{\Theta}_{SQ}, \boldsymbol{\nu}_Q, \boldsymbol{\psi}_Q, \boldsymbol{\phi}_Q, \boldsymbol{\eta}_Q \cdot \right. \\
&\left. \Phi_{\boldsymbol{\kappa}}\left(\kappa_{S,Q} | m_{\boldsymbol{\kappa}}, k_{\boldsymbol{\kappa}}\right) \Phi_{\boldsymbol{\pi}}(\pi_{S,Q} | m_{\boldsymbol{\pi}}, k_{\boldsymbol{\pi}}) \Phi_{\boldsymbol{\lambda}}(\lambda^*_{S,Q} | m_{\boldsymbol{\lambda}}, k_{\boldsymbol{\lambda}}) \Phi_{\boldsymbol{\nu}}(\nu_{S,Q})\right] \cdot \\
&\prod_{Q \in \mathbf{Q}} \left(\prod_{g \in \{\mathrm{E,M}\}} \Phi_{\boldsymbol{\psi}}(\check{\psi}_{\mathrm{g},Q}) \Phi_{\boldsymbol{\psi}}(\hat{\psi}_{\mathrm{g},Q}) \prod_{g \in \{\mathrm{F,E,M}\}} \Phi_{\boldsymbol{\phi}}(\check{\phi}_{g,Q}) \Phi_{\boldsymbol{\phi}}(\hat{\phi}_{g,Q}) \Phi_{\boldsymbol{\eta}}(\check{\eta}_{c,Q}) \Phi_{\boldsymbol{\eta}}(\hat{\eta}_{c,Q}) \right) \cdot \\
&\Psi^{(\boldsymbol{\kappa})}_m(m_{\boldsymbol{\kappa}}) \Psi^{(\boldsymbol{\kappa})}_k(k_{\boldsymbol{\kappa}}) \Psi^{(\boldsymbol{\pi})}_m(m_{\boldsymbol{\pi}}) \Psi^{(\boldsymbol{\pi})}_k(k_{\boldsymbol{\pi}}) \Psi^{(\boldsymbol{\lambda})}_m(m_{\boldsymbol{\lambda}}) \Psi^{(\boldsymbol{\lambda})}_k(k_{\boldsymbol{\lambda}})
\end{aligned} \tag{S44}$$

S1.6. USAMM Prior Construction

We implement a log-normal prior for $\kappa_{S,Q}$ (in eq. S43) because it is defined on the range $(0, \infty)$ which is the domain of a log-normal distribution. The choice was further motivated by our belief that $\kappa_{S,Q}$ should obtain its lowest probability at $\kappa_{S,Q} = 0$ or $\kappa_{S,Q} = \infty$. The same reasoning was applied to $\pi_{S,Q} \in (1, \infty)$, hence the use of a shifted log-normal distribution. For $\lambda^*_{S,Q}$, our choice of prior provided conjugacy, which allows for Gibbs sampling and hence fast computation (see S1.7).

Further, the hyperpriors for $\kappa_{S,Q}$, $\pi_{S,Q}$ and $\lambda^*_{S,Q}$ are in the three cases expressed as expectations on the prior parameters mean and coefficient of variation. To assign hyperpriors for $(m_{\boldsymbol{\kappa}}, m_{\boldsymbol{\pi}}, m_{\boldsymbol{\lambda}}, k_{\boldsymbol{\kappa}}, k_{\boldsymbol{\pi}}, k_{\boldsymbol{\lambda}})$, we first identify a plausible parameter range within which we believe with 95% certainty encapsulates the true (hyper)parameter value. Based on this range, we calculate the parameters used to define each distribution. Following this strategy, we chose a log-normal distribution with 95% of its density between 10km and 4,000 km as a prior for $m_{\boldsymbol{\kappa}}$. We motivate these values based on that 10 km would be considered as a short typical shipment distance whereas the upper limit is of the same order of magnitude as the coast to coast distance in the U.S and would thus correspond to a long distance. For $m_{\boldsymbol{\pi}}$, the choice is less straightforward, but our aim is to express our vague belief by allowing a wide range of shapes of the kernel. Due to the parameter range of $\pi_{S,Q}$, $(1, \infty)$, we chose a shifted log-normal hyperprior distribution and chose parameters such that $m_{\boldsymbol{\pi}}$ have 95% of its density between 2 and 1000. The lower value of $\pi_{S,Q} = 2$ corresponds to a kernel with steep slope, since the drop in kernel value from 50% to 5% of its value at distance zero would occur between distances κ and 2κ km. Conversely, the value of $\pi_{S,Q} = 1000$ corresponds to a fat-tailed kernel with the same decrease in kernel value at distances κ to 1000κ km. Our prior beliefs of the mean of $\lambda^*_{S,Q}$ (i.e. $m_{\boldsymbol{\lambda}}$) were expressed as a log-normal distribution with the interpretation that 95% of density is within 0.1 and 100 shipments per year for an average farm. This expectation is equivalent to a daily rate in the range

(0.00027,0.27) shipments per day. Note that other premises types are modeled relative to farms' shipping rates and may exhibit substantially higher rates. As in previous cases, the parameter choices here correspond to a wide range of plausible values and mirror our vague a priori beliefs regarding these values.

For the hyperprior distributions of ($k_{\boldsymbol{\kappa}}$, $k_{\boldsymbol{\pi}}$, $k_{\boldsymbol{\lambda}}$), we chose log-normal distributions due to the range on which they are defined and that the log-normal is of a form that assigns lowest density to values close zero and extremely large values. Large coefficient of variation indicates that the parameters have high heterogeneity between states and small values would indicate the opposite. To deduce parameters for this, we expressed our expectations on how similar the parameters are as a ratio between the mean and the 97.5th percentile of the corresponding $m_{\boldsymbol{\kappa}}, m_{\boldsymbol{\pi}}$ and $m_{\boldsymbol{\lambda}}$. We considered a value of two being reasonable as a lower limit, this is equivalent to a ratio of four between the 2.5th and 97.5th percentile. As upper limit we chose a value of ten which corresponds to a ratio of 100 between the 2.5th and 97.5th percentile. If the mean of κ_S (i.e. $m_{\boldsymbol{\kappa}}$) is estimated at 100 km. The interpretation of the hyperprior would be that limits 2 and 10 correspond to distributions of κ with 95% of its density in the range $(50, 200)$ km and $(10, 1000)$ km, respectively. From the reasoning above, hyperpriors $\Psi_k^{(\kappa)}(k_{\boldsymbol{\kappa}})$, $\Psi_k^{(\pi)}(k_{\boldsymbol{\pi}})$ and $\Psi_k^{(\lambda)}(k_{\boldsymbol{\lambda}})$ were implemented as log-normal distributions with 95% of the density between 0.3650 and 1.724.

Parameters $\boldsymbol{\psi}$, $\boldsymbol{\phi}$ and $\boldsymbol{\eta}$ were modeled as shared across the U.S. These parameters, and also $\boldsymbol{\nu}$, were modeled without a hierarchical structure with priors

$$\begin{aligned}
\nu_{S,Q} &\sim \text{log-normal}_{95\%}(0.01, 100), \\
\check{\psi}_{g,Q} &\sim \text{Half-Cauchy}\,(1)\,, \\
\hat{\psi}_{g,Q} &\sim \text{Half-Cauchy}\,(1)\,, \\
\check{\phi}_{g,Q} &\sim \text{N}_{95\%}\,(0,1)\,, \\
\hat{\phi}_{g,Q} &\sim \text{N}_{95\%}\,(0,1)\,, \\
\check{\eta}_{c,Q} &\sim \text{N}_{95\%}\,(-1,1)\,, \\
\hat{\eta}_{c,Q} &\sim \text{N}_{95\%}\,(-1,1)\,,
\end{aligned} \tag{S45}$$

where $\text{log-normal}_{95\%}\,(l,u)$ and $\text{N}_{95\%}\,(l,u)$ denote a log-normal and a normal distribution, respectively, with 95% of its density within the range (l,u). As prior for $\nu_{S,Q}$, denoted $\Phi_{\boldsymbol{\nu}}(\nu_{S,Q})$, we let our vague beliefs of the propensity to attract shipments be represented by the a log-normal distribution with 95% of its density within the range $(0.01, 100)$. The interpretation of this is that we believe with 95% certainty that the parameter falls within the wide range from 0.01 to 100 times the propensity to attract shipments for our reference state (Iowa). For $\check{\phi}_{g,Q}$ and $\hat{\phi}_{g,Q}$, the range $(0,1)$ were based on our prior beliefs that the number of in-going or out-going shipments is unlikely to scale hyperlinearly or negatively with premises size. Yet, we do not exclude these possibilities and permit estimates outside of this range, should the data strongly deviate from our a priori assumption. Our prior beliefs for the parameters $\check{\psi}_{\text{E},Q}$, $\hat{\psi}_{\text{E},Q}$, $\check{\psi}_{\text{M},Q}$ and $\hat{\psi}_{\text{M},Q}$ in S45 are expressed through a Half-Cauchy Distribution with scale equal to one. The Half-Cauchy distribution supports values ranging from zero to infinity and we chose this distribution due to that it is a wide distribution and therefore mirrors our vague beliefs regarding these parameters. Finally, we chose the prior distribution for $\check{\eta}_{c,Q}$ and $\hat{\eta}_{c,Q}$ as a Normal distribution with 95% of the density between minus one and one. This symmetric distribution does not favor positive nor negative values; we

give equal a priori weight to increased and decreased out- and/or incoming shipments with higher value of each covariate.

S1.7. USAMM Computation

Since eq. S44 is not of a standard form, it is not possible to make random draws from the posterior directly and we must rely on numerical methods. We therefore used a Markov Chain Monte Carlo (MCMC) algorithm to sample from the joint posterior of all model parameters. The idea behind this is to create a Markov Chain with limiting state distribution equal to the posterior and from there obtain samples from the posterior distribution. Details on MCMC can be found in e.g. [6].

In practice, we did not use the likelihood (eq. S42) with factors of the form as expressed in eq. S41. Since, there have been no observed shipments for the majority of combinations of origin- and destination counties and days, most of the $|\mathbf{Y}_{\omega,\delta,\tau}|$ in eq. S41 are equal to zero, which means a simplification of the expression can speed up calculations. The corresponding terms in the multinomial distribution are thus equal to 1 and can therefore be disregarded. As opposed to [4], we here take not only state level differences into account but also seasonality and we therefore split the likelihood (S42) into a product of state and season specific terms as

$$\begin{aligned}&L_{\mathbf{Y}}\left(\mathbf{Y}|\boldsymbol{\Theta},\boldsymbol{\lambda},\boldsymbol{\nu},\boldsymbol{\psi},\boldsymbol{\phi},\boldsymbol{\eta}\right)=\\&\prod_{Q\in\mathbf{Q}}\prod_{S\in\mathbf{U}}P_{S,Q}\left(\mathbf{Y}_{S,Q}|\boldsymbol{\Theta}_{S,Q},\lambda^{*}_{S,Q},\boldsymbol{\nu}_{Q},\boldsymbol{\psi}_{Q},\boldsymbol{\phi}_{Q},\boldsymbol{\eta}_{Q}\right)\propto\\&\prod_{Q\in\mathbf{Q}}\prod_{S\in\mathbf{U}}\left(\text{Poisson}\left(|\mathbf{Y}_{S,Q}|\middle|\tilde{\lambda}_{S,Q}\times|\boldsymbol{\tau}_{Q}|\times r_{S,Q}\right)\prod_{y\in\mathbf{Y}_{S}}\left(\frac{p_{(\delta_{y}|\omega_{y}),Q}}{q_{(\delta_{t}|\omega_{t}),Q}}\right)\right.\end{aligned}\tag{S46}$$

That is, the multinomial distribution is expressed as a product, where every factor corresponds to one observed transport. The interpretation of eq. S46 is the probability of observing $|\mathbf{Y}_{S,Q}|$ transports from state $S\in\mathbf{U}$ in quarter Q, multiplied with the probabilities of the observed shipments $y,\forall y\in\mathbf{Y}_{S,Q}$ having destination $\delta\in\bar{\mathbf{V}}_{S}$ and origin $\omega\in\mathbf{V}_{S}$ conditional on the transports leaving the state.

With the exception of $\boldsymbol{\lambda}$, where the choice of prior provided conjugacy, conditional distributions are not of standard form. We therefore used Gibbs sampling for $\boldsymbol{\lambda}$ and implemented Metropolis-Hastings updates [7] of all other parameters to obtain samples from the posterior distribution (eq. S44). For this, we created a Markov Chain with transition distribution $\Pi(y_{\text{prop}}|y_{\text{acc}})$ where y_{prop} and y_{acc} denotes the proposed and current state of the Markov Chain respectively. This transition distribution is created as a product of the proposal distribution $Q(y_{\text{prop}}|y_{\text{acc}})$ from which new parameter values are proposed and an acceptance ratio $\Upsilon\left(y_{\text{prop}}|y_{\text{acc}}\right)$ that specifies the probability of accepting the proposed value as a sample drawn from the posterior distribution. By choosing $\Upsilon\left(y_{\text{prop}}|y_{\text{acc}}\right)$ to be

$$\Upsilon(\mathrm{y}_{prop}|y_{acc})=\min\left(1,\frac{Q(y_{acc}|y_{prop})P_{\mathbf{T}}(y_{prop})}{Q(y_{prop}|y_{acc})P_{\mathbf{T}}(y_{acc})}\right),\tag{S47}$$

the transition distribution

$$\Pi(y_{\text{prop}}|y_{\text{old}})=Q(y_{\text{prop}}|y_{\text{acc}})\Upsilon\left(y_{\text{prop}}|y_{\text{acc}}\right)\tag{S48}$$

will have its limiting state distribution equal to the target density $P_{\mathbf{Y}}$, which is the posterior distribution (eq. S44).

We updated $\kappa_{S,Q}$ and $\pi_{S,Q}-1$ jointly with a bivariate normal random walk on the log scale (i.e. we proposed $ln(\kappa_{S,Q})$ and $ln(\pi_{S,Q}-1)$ from a bivariate normal distribution). For the parameters ν, we used a multivariate normal random walk on the log scale in 47 dimensions. Here, $\nu_{\text{Iowa},Q}=1$ was excluded since it was fixed and used as reference. Parameters $\psi_{\text{E},Q}$ and $\psi_{\text{M},Q}$, modeling the effect of premises types, were proposed jointly on the log-scale, using a multivariate normal distribution. That is, the random walk was performed on the parameters $(\phi_{\text{F},Q},\phi_{\text{E},Q},\phi_{\text{M},Q},ln(\psi_{\text{E},Q}),ln(\psi_{\text{M},Q}),\eta_{1,Q},\eta_{2,Q},\eta_{3,Q})$ for both parameters governing sending and receiving, and here, $\psi_{\text{F},Q}$ is excluded since it is used as reference for $\psi_{\text{E},Q}$ and $\psi_{\text{M},Q}$. All hyperparameters (m_x,k_x) were proposed from a bivariate normal distribution on the log scale. For the parameters proposed from multivariate normal distributions, we implement an optimized version of the Robbins-Monro algorithm introduced in [8], to facilitate good mixing. This involves tuning the covariance of the proposal distribution and a scaling factor so that the MCMC algorithm reaches a long term acceptance ratio equal to a predefined value, here set to 0.234 as proposed in [9]. Since the choice of prior for $\boldsymbol{\lambda}$ provided conjugacy, we were able to implement Gibbs-sampling and sampled directly from their conditional distributions

$$\lambda^*{}_{S,Q}\sim\text{Gamma}\left(\alpha_\lambda|\mathbf{Y}_{S,\boldsymbol{\tau}_Q}|,\beta_\lambda+|\boldsymbol{\tau}|r_{S,Q}\right). \tag{S49}$$

Some of the parameters exhibited a bimodal structure in the corresponding marginal posterior distributions, which provided poor mixing using the technique described in [8]. To facilitate good mixing for these parameters, we made preliminary runs to identify the two high density regions (HDR) and assigned the accepted parameters from the MCMC chains to one of the two HDRs. Based on the samples in each partitioning, we calculated the sample mean and covariance matrix and multiplied the latter by four to obtain reasonably wide proposal distributions for each HDR. To create a proposal distribution from the estimation of the two HDRs, we proposed from the two distributions with probability proportional to the number of samples in each HDR in the preliminary runs. Note that this technique does not affect the ergodicity or change the limiting state distribution of the Markov Chain, but only provides an ad-hoc solution to circumvent the poor mixing for the parameters with bimodal marginal posteriors.

We performed nine runs of the MCMC algorithm for beef and dairy and each run consisted of 750,000 iterations in which we discarded 50,000 as burn in. To avoid large output files, we thinned our sampler and kept every 100th sample. We implemented over-dispersed seeding of parameters to assert convergence to the same region in every implementation, which was evaluated through Potential Scale Reduction Factor (PSRF). We also estimated effective sample size [10].

S1.8. Shipment simulation

USAMMv2.1 was used within USDOS to simulate shipments from infected premises every timestep (day) τ. This was done by first simulating the number of shipments leaving each county ω in which there were infected premises as a Poisson random variable $Y^*_{\omega,\tau}$ according to equation (S15). Second, the shipments were distributed among all counties by sampling from

a multinomial distribution with $n = Y^*_{\omega,\tau}$ and a vector of probabilities with elements given by equation (S23), so that each destination county δ was assigned a number of shipments $Y^*_{\omega,\delta,\tau}$ arriving from ω (see supplemental material equation S40). Third, origin premises for the $Y^*_{\omega,\delta,\tau}$ shipments between ω and δ were sampled from the premises population of ω as a multinomial random variable with $n = Y^*_{\omega,\delta,\tau}$, and a vector of probabilities P with elements associated with each premises and proportional to the individual weights in equation (S24):

$$P_i = \hat{\psi}_{g(i),Q(\tau)} \left(\frac{h_i}{\bar{h}_{g(i)}} \right)^{\hat{\phi}_{g(i),Q(\tau)}}, \tag{S50}$$

where $g(i)$ is the type of premises i. If the sampled origin premises was one of the infected premises, a destination premises was also sampled from among the population of premises in δ in a similar fashion, but from a categorical distribution (i.e. a multinomial distribution with $n = 1$) with weights associated with the individual premises in δ according to

$$P_i = \check{\psi}_{g(i),Q(\tau)} \left(\frac{h_i}{\bar{h}_{g(i)}} \right)^{\check{\phi}_{g(i),Q(\tau)}}. \tag{S51}$$

In order to simulate shipments corresponding to a complete shipment network, ϵ_u was set to 1.0 for all states u for the purpose of simulating shipments within USDOS. Further, the reason for only simulating shipments from infected premises was that in USDOS shipment from non-infected premises has no effect on transmission dynamics whatsoever, and omitting this step for all counties and premises where no infection was present avoided a considerable amount of unnecessary calculations.

S2. Supplemental Results

S2.1. Partial Transition

The maximum infectious period affects both how long an individual premises remains infectious, and how long an individual simulation takes to run. We examined the run time and outbreak metrics of the 15, 20, and 30-day cutoff points to balance computational efficiency with accurately capturing disease dynamics. As expected, we find that increasing the maximum infectious period cut-off increases the run time length. A single base scenario replicate without partial transition took on average 10.61 seconds, a base scenario replicate with 15-day cutoff took on average 24.3 seconds, 20-day cutoff took on average 29.71 seconds, and 30-day cutoff took on average 34.77 seconds (Table S3). The standard deviation around single replicate run times also increased as the cutoff period increased.

Table S3: Run times for USDOSv2.1 base scenario for three different maximum infectious period lengths. The single replicate lengths show single simulations seeded in one of the 3049 counties.

		**Partial Transition**		
		**Maximum Infectious Period**		
	**No Partial Transition**	**15 Day**	**20 Day**	**30 Day**
Mean single replicate length (seconds)	10.61	24.30	29.71	34.77
Standard deviation of single replicate length (seconds)	47.48	116.18	143.35	167.29

Our results show that the predicted distribution of outbreak duration differs substantially between the 15 day cut-off scenario and the other two scenarios (Figures S4 & S5). Specifically, outbreak duration is predicted to be shorter in the 15 day cut-off scenario. This difference is most clearly seen in the northern plains regions in the median outbreak maps; the 20 and 30 day cut-off scenarios predict longer duration than the 15 day cut-off scenario in this region (Figures S5a versus S5b & S5c). These results also show that outbreak duration predictions from the 20 and 30 day cut-off scenarios are similar, and do not show the marked differences seen when comparing to the 15 day cut-off scenario.

Similar to the duration results, the total number of infected premises results from the 20 and 30 day cut-off scenarios have few differences (Figures S6). Additionally, the 15 day cut-off scenario results for total infected premises are much more similar to the 20 and 30 day cut-off scenarios than the results for duration (Figure S6).

Overall, we find that the variation in predicted outbreaks is much lower for total infected premises than for outbreak duration (Figures S4 & S6). This finding is consistent with the expected base scenario outcome of adding partial transition. The scaling of the partial transition function ensured that the overall level of transmission was the comparable between USDOSv2.0 and USDOSv2.1. Since transmission rates were informed by data (see [1] for details), the scaling of the partial transition function prevented the addition of internal dynamics of premises in USDOS from artificially amplifying spread. Therefore, since the scaling factor s accounts for the maximum infectiousness cut-off it is unsurprising that the results from the total infected premises are similar among the three scenarios (Figure S6). Similarly, the predicted outbreak duration should get longer when the maximum infectious period value is increased; a pattern we see when comparing the three different cut-off scenario results (Figure S4). However, the predicted outbreak duration changes very little between the 20 and 30 day cut-off scenarios, suggesting that the increase in maximum infectious value in this range has little effect on the outbreak dynamics.

The similarity between the 20 and 30 day cut-off scenario results for both outbreak duration and infectious period, are consistent with the shape of the partial transition curve,

which begins to plateau around 15 days (Figure S3). Our results suggest that a day 15 cut-off is not sufficiently long enough to capture the within-herd outbreak trajectory. However, a 20 day cut-off produces similar results to a 30 day cut-off across multiple outbreak trajectories. Additionally, the 20 day cut-off scenario had shorter run times, making it both long enough to capture the outbreak dynamics and short enough to be manageable from a computational perspective. Based on these results, the default maximum infectious period in USDOSv2.1 is 20 days (Table S1).

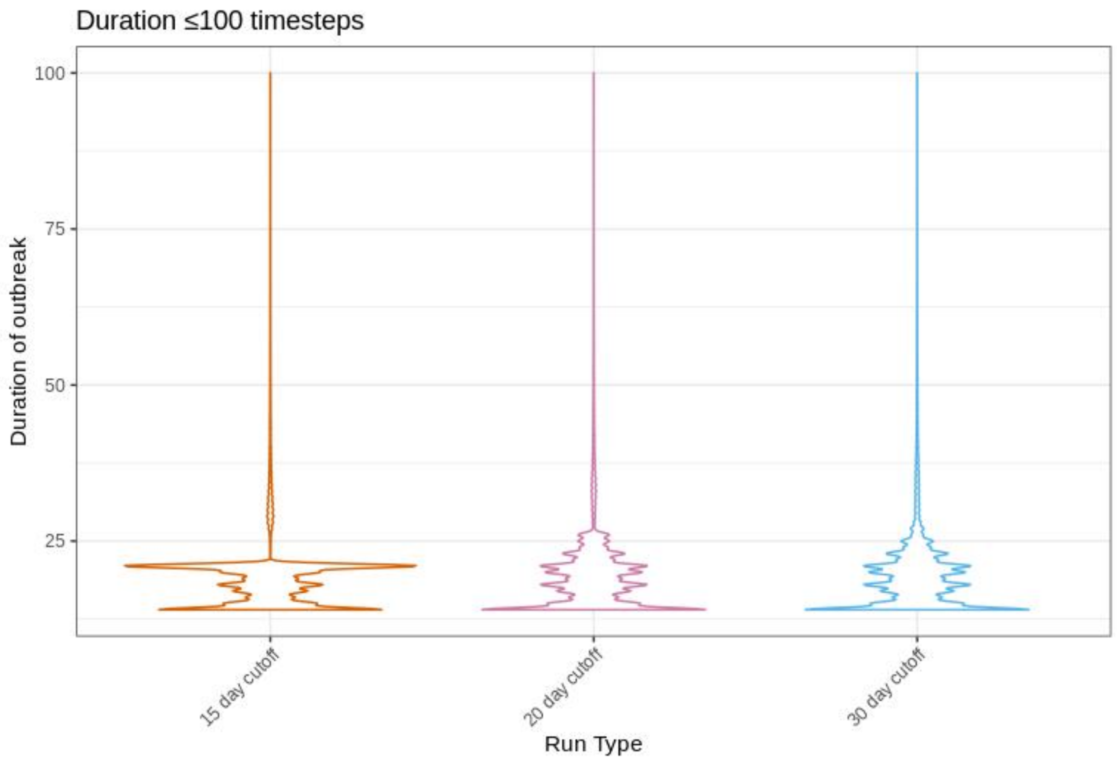

(a)

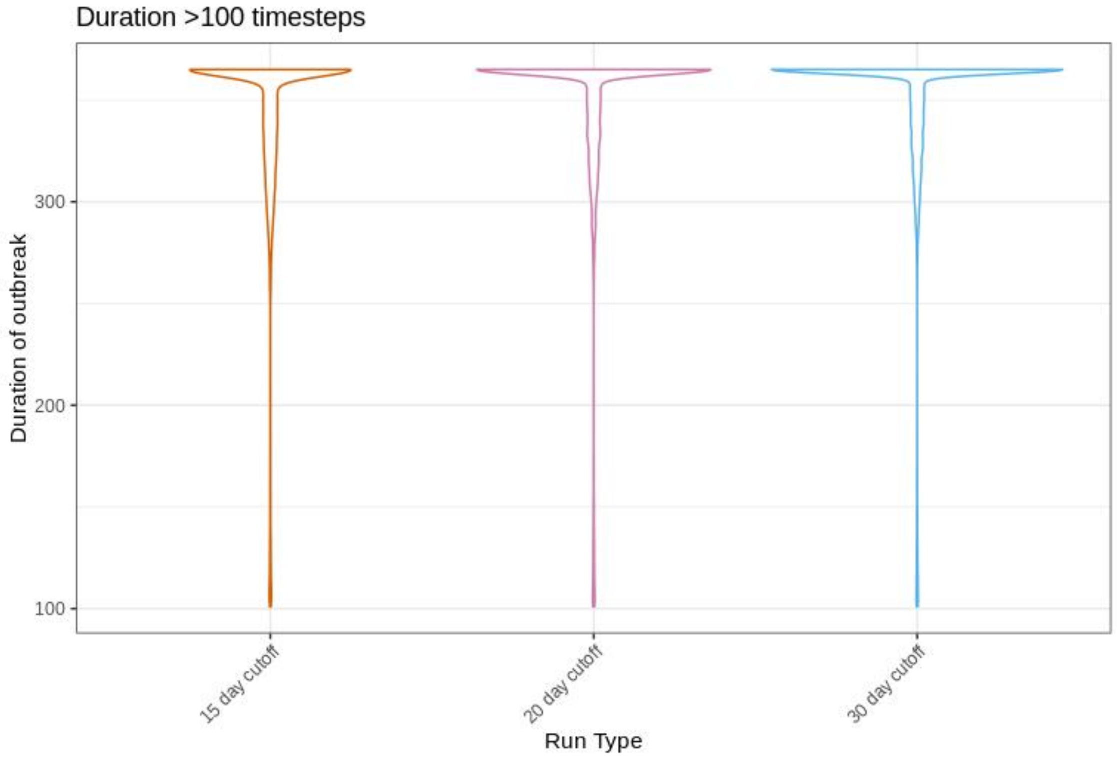

(b)

Figure S4: Outbreak duration for base scenario simulations with three partial transition maximum infectious period cut-off points. Distributions of duration of (a) less than 100 days and (b) greater than 100 days for simulations with a maximum infectious period of 15 days (orange, left), 20 days (purple, center) and 30 days (blue, right).

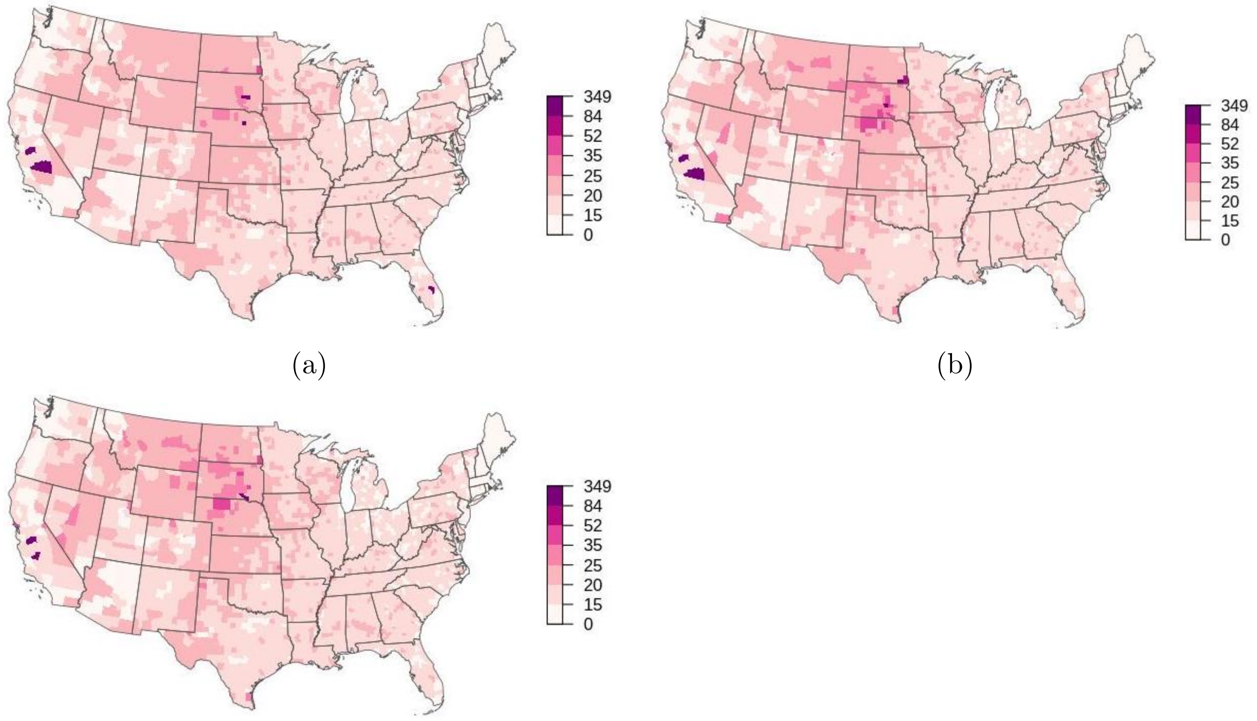

(a)

(b)

(c)

Figure S5: Median outbreak duration when infection is seeded in each county, summarized across 304,900 simulations for each map with a maximum infectious period of (a) 15 days, (c) 20 days and (c) 30 days.

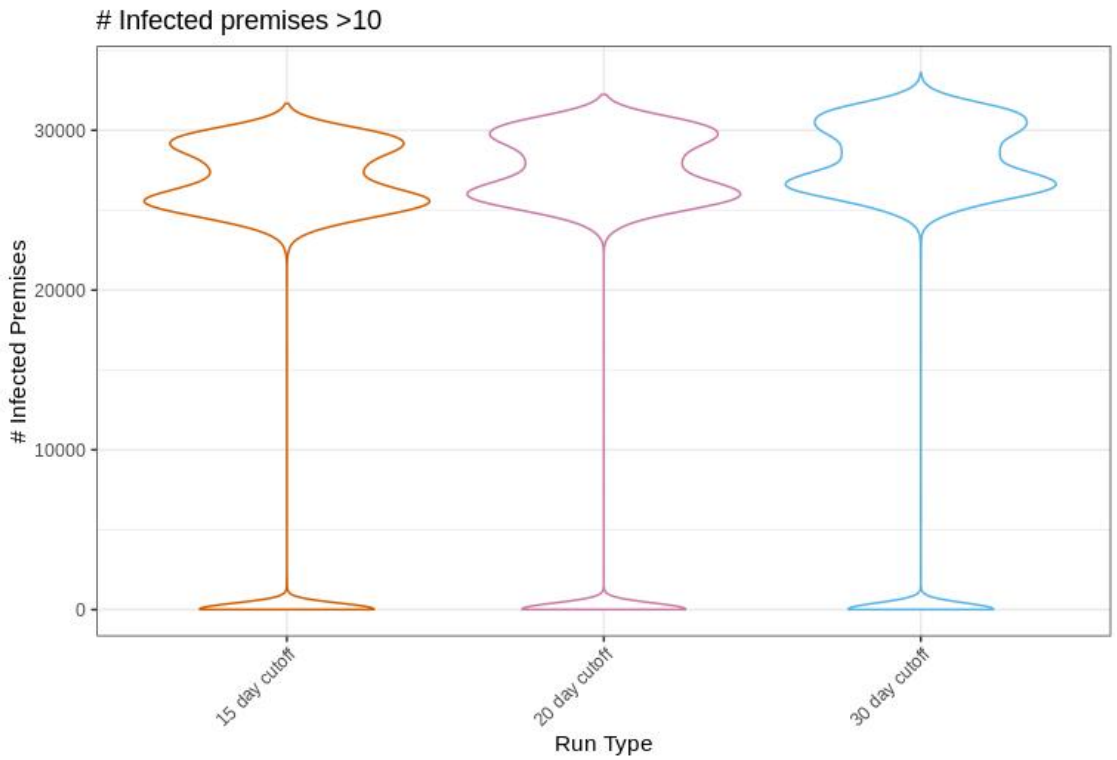

Figure S6: Distribution of number of infected premises for base scenario simulations with more than 10 infected premises and with a maximum infectious period of 15 days (orange, left), 20 days (purple, center) and 30 days (blue, right).

Table S4: Comparison between median and upper 97.5th percentile of outbreaks for base scenario without partial transition and with partial transition.

	**Duration**		**Number of Premises Infected**		**Number of Counties Infected**	
**Model Scenario**	**Median**	**Upper 97.5th**	**Median**	**Upper 97.5th**	**Median**	**Upper 97.5th**
Base scenario without partial transition	13	258	1	23877	1	1459
Base scenario with partial transition	19	358	1	26334	1	1606

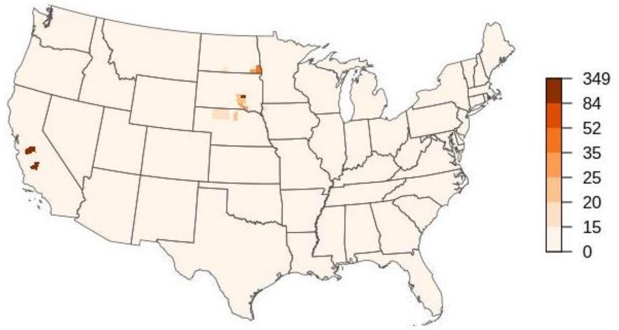

(a) without partial transition

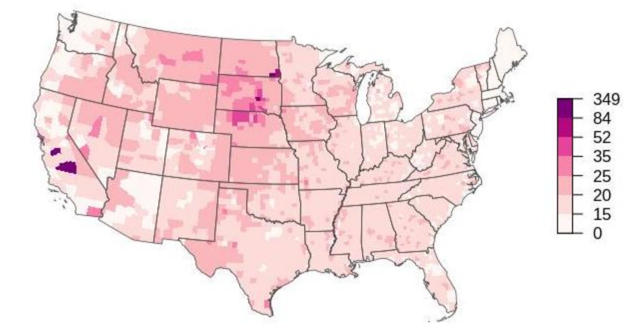

(b) with partial transition

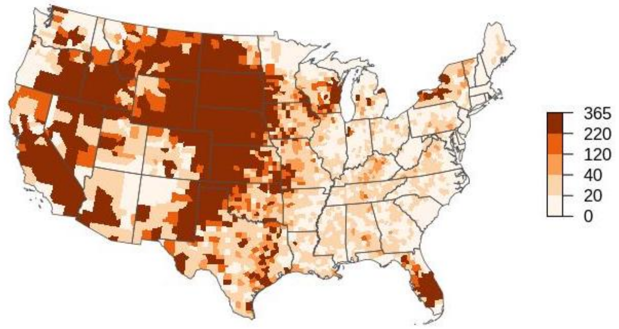

(c) without partial transition

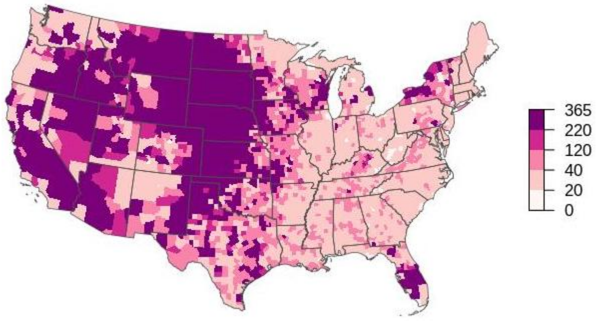

(d) with partial transition

Figure S7: (a,c) Median and (b,d) upper 97.5th percentile outbreak duration when infection is seeded in each county, summarized across 304,900 simulations for each map. (a,b) show the base scenario without partial transition and (c,d) show the base scenario with partial transition. Panel (b) is the same map as in Figure S5b.

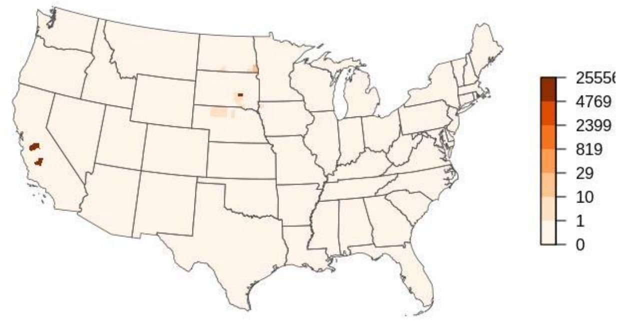

(a) without partial transition

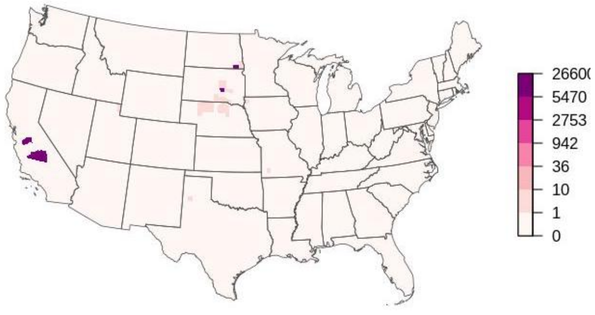

(b) with partial transition

Figure S8: (a,c) Median number of infected premises when infection is seeded in each county, summarized across 304,900 simulations for each map. (a) shows the base scenario without partial transition and (b) shows the base scenario with partial transition.

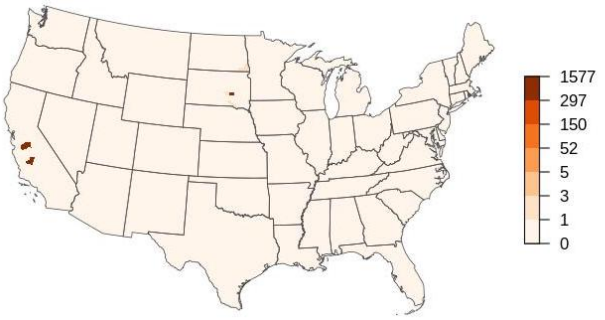

(a) without partial transition

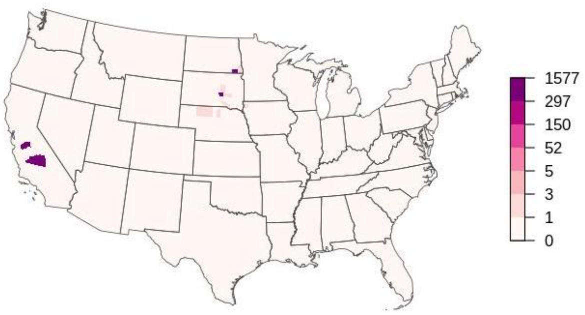

(b) with partial transition

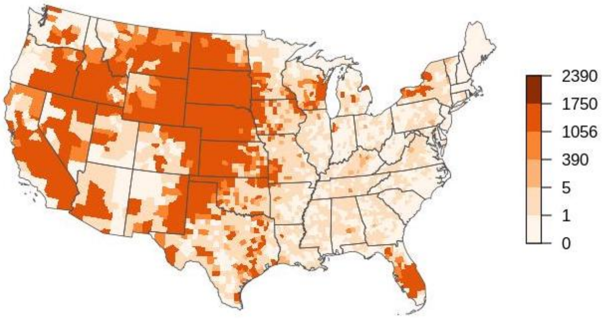

(c) without partial transition

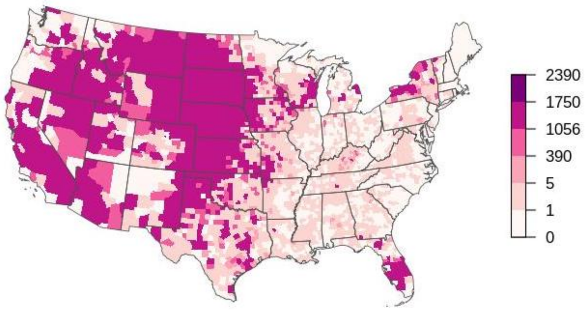

(d) with partial transition

Figure S9: (a,c) Median and (b,d) upper 97.5th percentile number of counties infected when infection is seeded in each county, summarized across 304,900 simulations for each map. (a,b) show the base scenario without partial transition and (c,d) show the base scenario with partial transition.

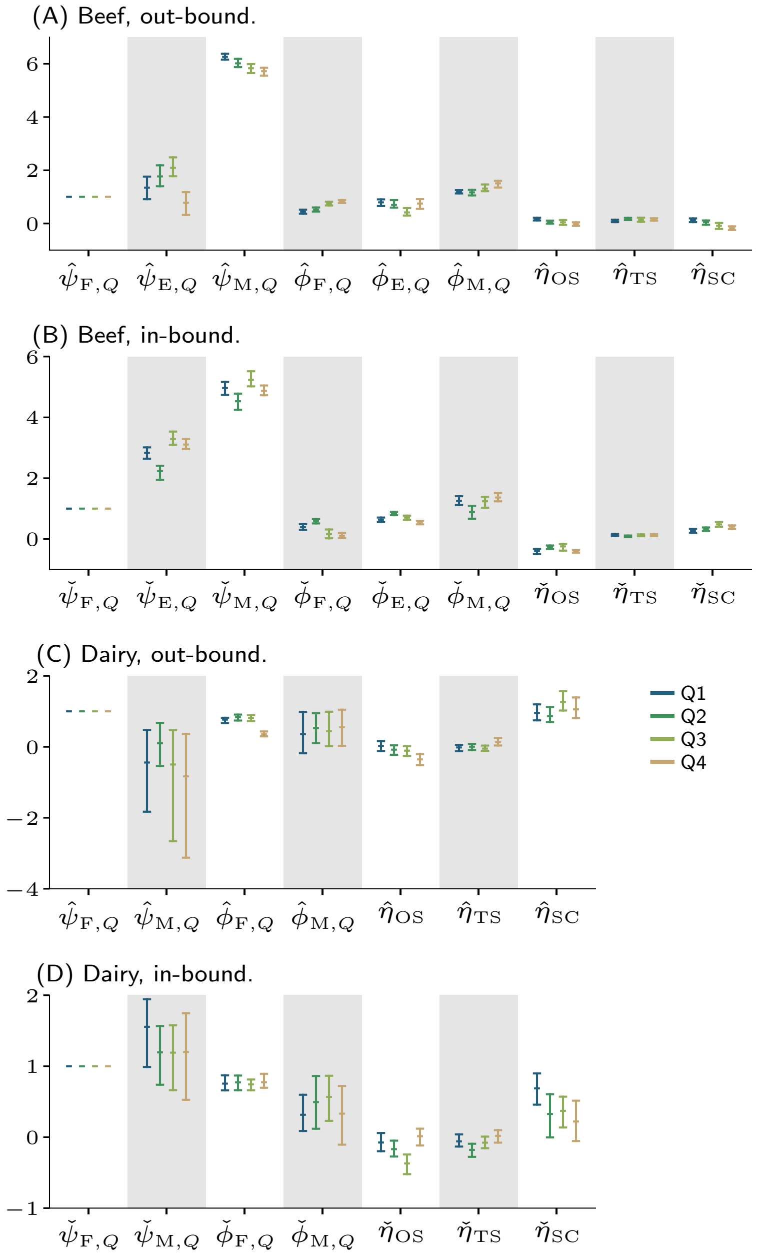

Figure S10: Median and 95% central credibility intervals of posterior estimates of USAMMv2.1 parameters modeling effects on shipment rate exclusive to the refined version. Parameters are coefficients (ψ) and exponents (ϕ) of the power law relationships between herd size and premises weight, for premises types farm (F), feedlot (E) and market (M); as well as scaling exponents (η) for the industry covariates *operations with sales* (OS), *total sales in head* (TS) and *slaughter connectivity* (SC). Estimates for the quarters (Q) of the year indicated by colors with the leftmost estimate in each group representing Q1 and the following quarters in sequence to the right. Estimates for $\eta_{F,Q}$ for both directions and commodities were fixed at 1.0 in the model.

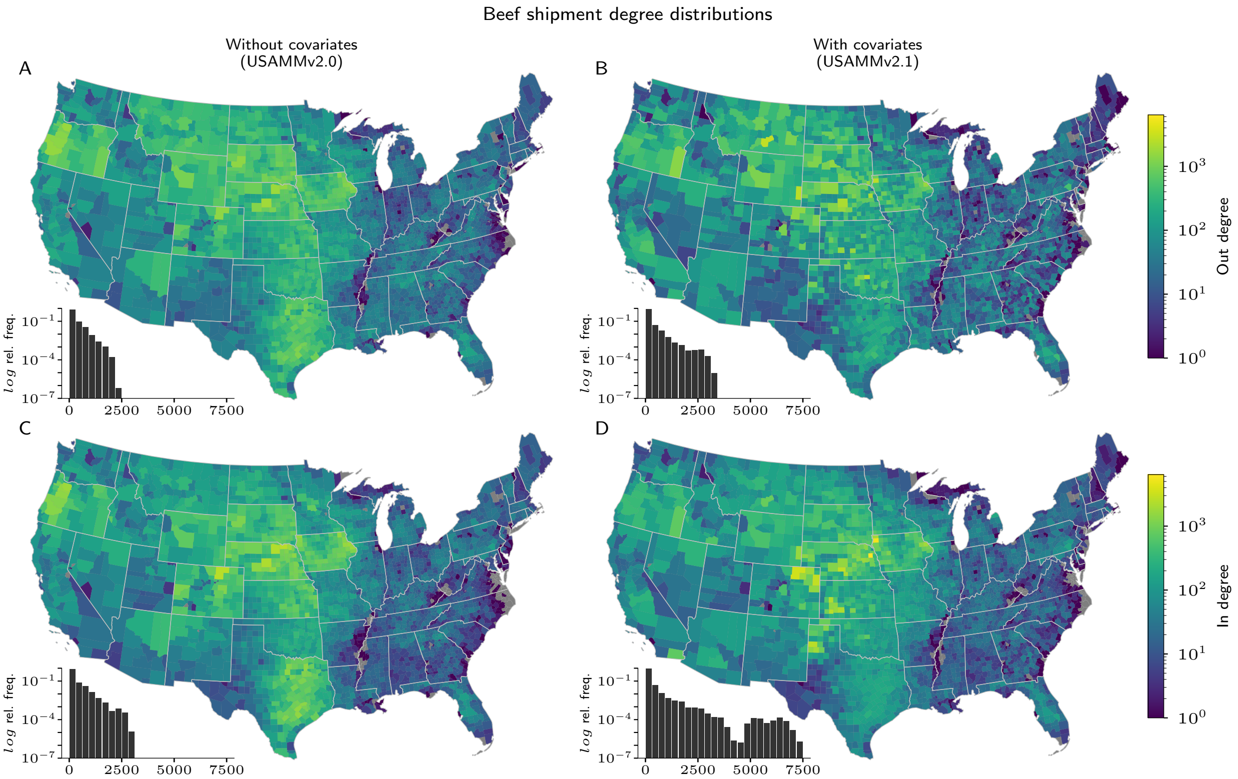

Figure S11: County-level degree distributions of logged outgoing (A and B) and incoming (C and D) beef shipments for the simplified USAMM implementation (A and C), and the refined implementation (B and D). The simplified implementation excludes effects of county industry covariates and premises size scaling while the refined includes such effects. The maps show the median degree of each county across 1,000 replicate beef networks; the inset histograms illustrate the logged relative frequency distribution of degree across all counties in all 1,000 replicates.

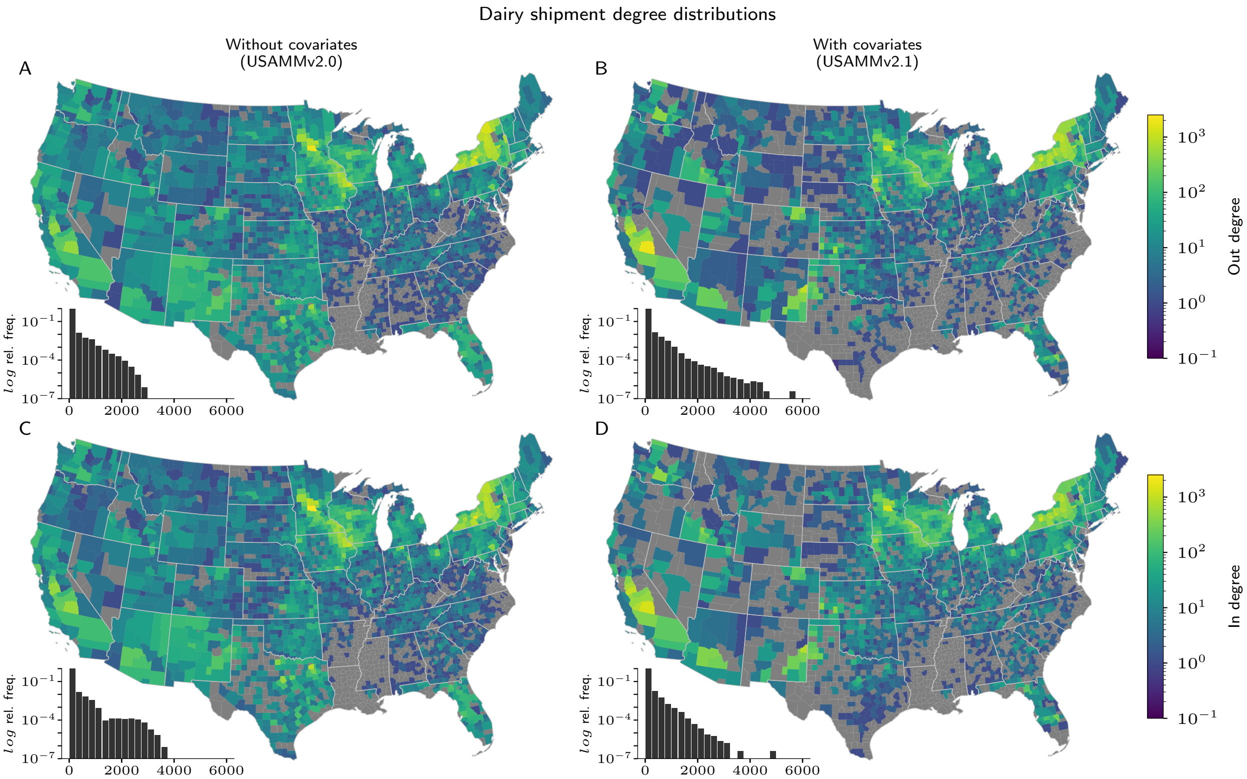

Figure S12: County-level degree distributions of logged outgoing (A and B) and incoming (C and D) dairy shipments for the simplified USAMM implementation (A and C), and the refined implementation (B and D). The simplified implementation excludes effects of county industry covariates and premises size scaling while the refined includes such effects. The maps show the median degree of each county across 1,000 replicate dairy networks; the inset histograms illustrate the logged relative frequency distribution of degree across all counties in all 1,000 replicates.

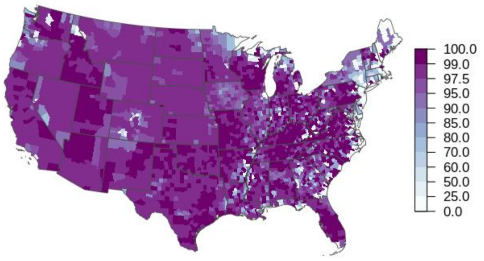

(a) IP cull, DC vacc. run with USAMMv2.1 simple

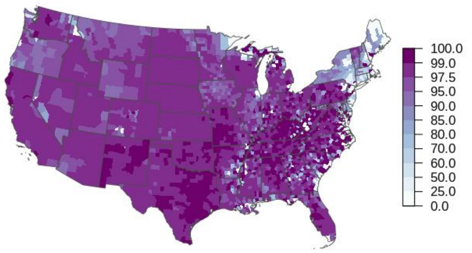

(b) IP cull, DC vacc. run with USAMMv2.1 refined

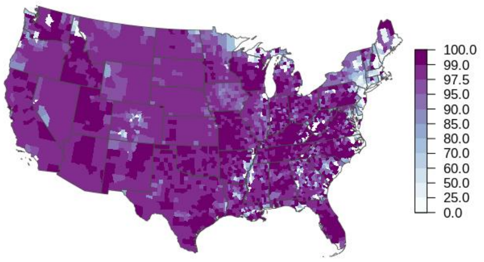

(c) IP cull 10km ring vacc. run with USAMMv2.1 simple

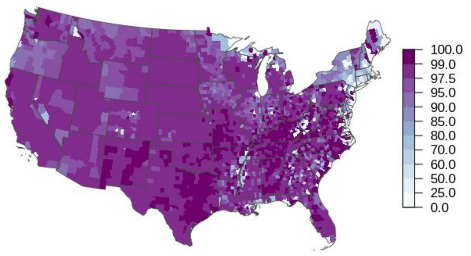

(d) IP cull 10km ring vacc. run with USAMMv2.1 refined

Figure S13: The proportion of local transmission when infection is seeded in each county, summarized across 304,900 simulations for each map. (a,b) show the IP cull and DC vaccination scenario, (c,d) show the IP cull and 10 km ring vaccination scenario. (a,c) show results from USDOS run with run with USAMMv2.1 simple (b,d) show results from USDOS run with run with USAMMv2.1 refined.

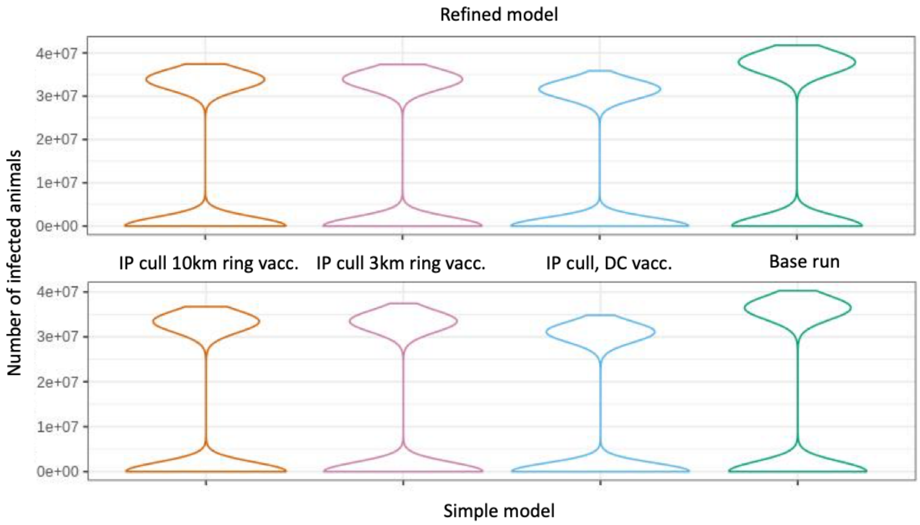

Figure S14: Number of animals infected > 1000 across each scenario, comparing our simple and refined model results.

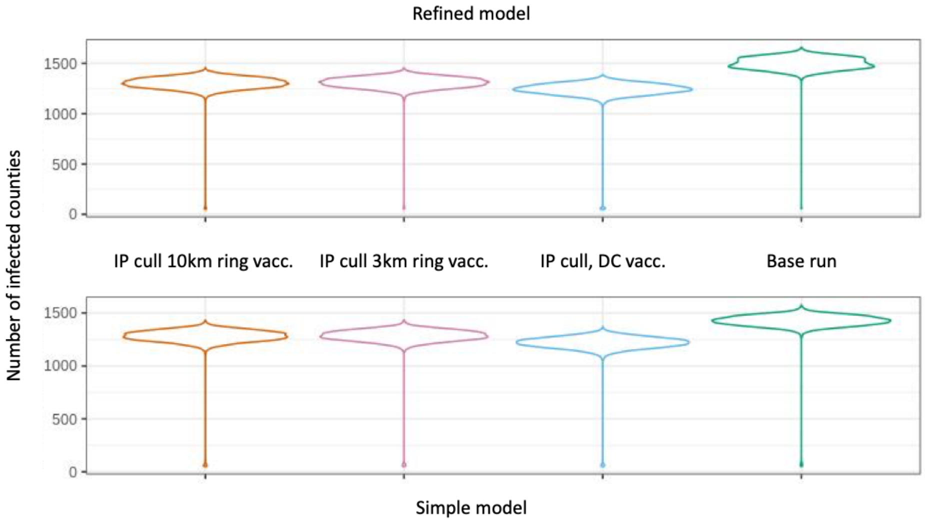

Figure S15: Number of counties infected > 50 across each scenario, comparing our simple and refined model results.

Table S5: Comparison between median and upper 97.5th percentile of outbreaks for each control scenario using the simple and refined models.

	Duration		Number of Premises Infected		Number of Counties Infected	
**Model Scenario**	**Median**	**Upper 97.5th**	**Median**	**Upper 97.5th**	**Median**	**Upper 97.5th**
Base scenario, simple	13	258	1	23877	1	1459
Base scenario, refined	13	259	1	23167	1	1385
3km ring vaccination, simple	13	202	1	18665	1	1255
3km ring vaccination, refined	13	198	1	18084	1	1220
10km ring vaccination, simple	13	201	1	18567	1	1249
10km ring vaccination, refined	13	198	1	18014	1	1215
DC vaccination, simple	13	180	1	15976	1	1183
DC vaccination, refined	13	170	1	14942	1	1127

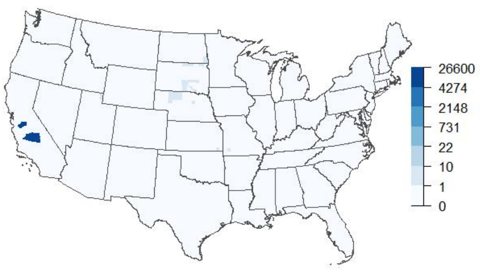

(a) USDOS with USAMMv2.1 simple

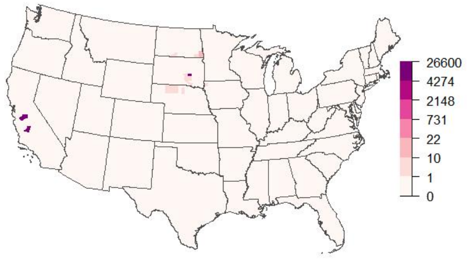

(b) USDOS with USAMMv2.1 refined

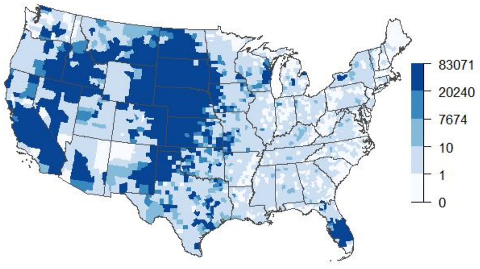

(c) USDOS with USAMMv2.1 simple

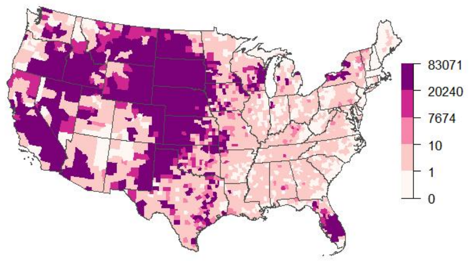

(d) USDOS with USAMMv2.1 refined

Figure S16: (a,c) Median and (b,d) upper 97.5th percentile number of infected premises when infection is seeded in each county, summarized across 304,900 simulations for each map. (a,b) show the base scenario run with USAMMv2.1 simple and (c,d) show the base scenario run with USAMMv2.1 refined.

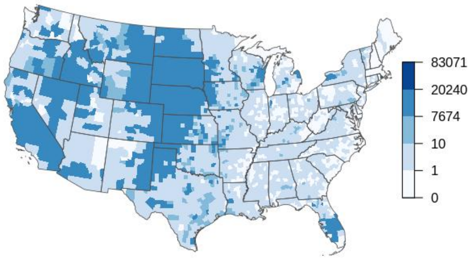

(a) IP cull, DC vacc. run with USAMMv2.1 simple

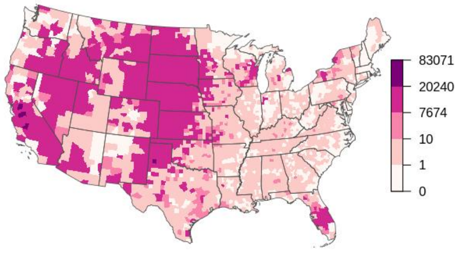

(b) IP cull, DC vacc. run with USAMMv2.1 refined

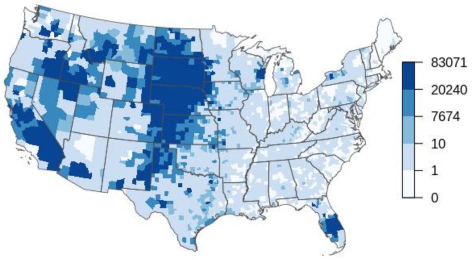

(c) IP cull 3km ring vacc. run with USAMMv2.1 simple

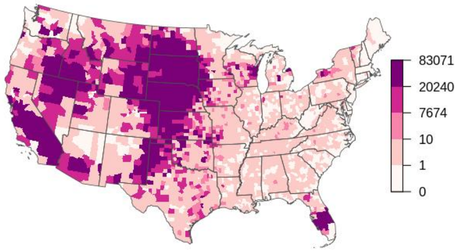

(d) IP cull 3km ring vacc. run with USAMMv2.1 refined

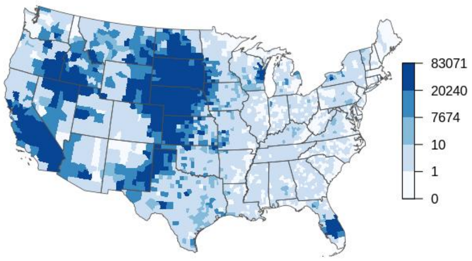

(e) IP cull 10km ring vacc. run with USAMMv2.1 simple

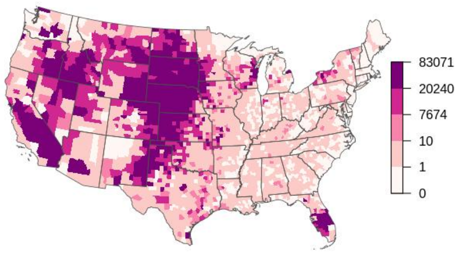

(f) IP cull 10km ring vacc. run with USAMMv2.1 refined

Figure S17: The upper 97.5th percentile number of infected premises when infection is seeded in each county, summarized across 304,900 simulations for each map. (a,b) show the IP cull and DC vaccination scenario, (c,d) show the IP cull 3 km ring vaccination scenario and (e,f) show the IP cull and 10 km ring vaccination scenario. (a,c,e) show results from USDOS run with run with USAMMv2.1 simple (b,d,f) show results from USDOS run with run with USAMMv2.1 refined.

S2.3. Sensitivity Analysis

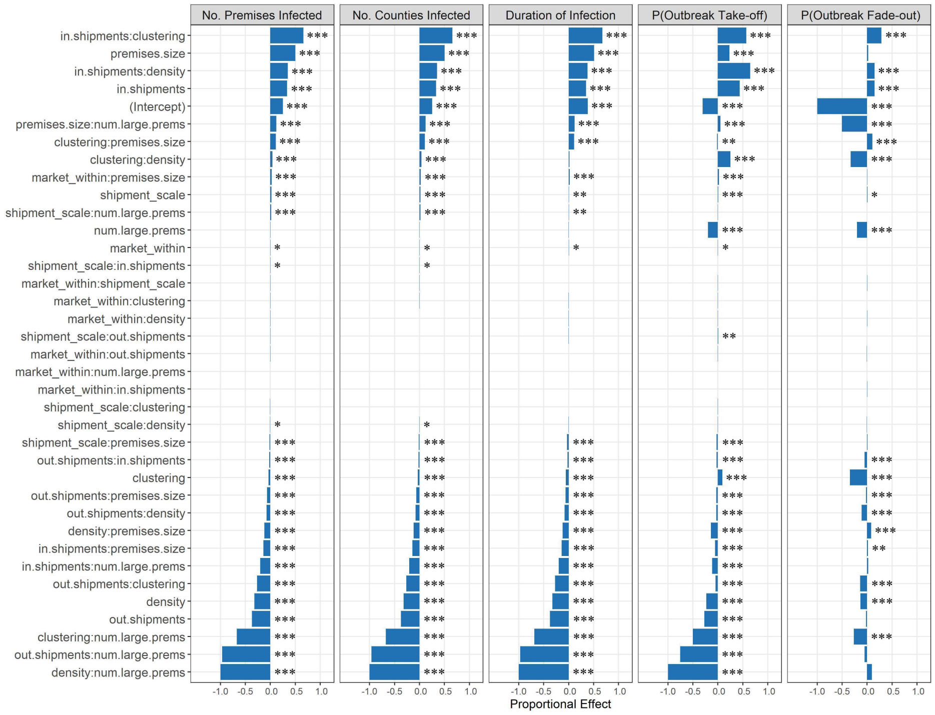

Figure S18: Full sensitivity results for each model. Symbol * indicates significance at $p = 0.05$, ** indicates significance at $p = 0.01$, and *** indicates significance at $p = 0.001$.

Supplemental References

[1] K. Tsao, S. Sellman, L. M. Beck-Johnson, D. J. Murrieta, C. Hallman, T. Lindström, R. S. Miller, K. Portacci, M. J. Tildesley, C. T. Webb, Effects of regional differences and demography in modelling foot-and-mouth disease in cattle at the national scale, Interface Focus 10 (2019). doi:10.1098/rsfs.2019.0054.

[2] L. M. Beck-Johnson, E. E. Gorsich, C. Hallman, M. J. Tildesley, R. S. Miller, C. T. Webb, An exploration of within-herd dynamics of a transboundary livestock disease: a foot and mouth disease case study, In Review.

[3] G. A. Dewell, C. A. Simpson, R. D. Dewell, D. R. Hyatt, K. E. Belk, J. A. Scanga, P. S. Morley, T. Grandin, G. C. Smith, D. A. Dargatz, B. A. Wagner, M. D. Salman, Risk associated with transportation and lairage on hide contamination with Salmonella enterica in finished beef cattle at slaughter, Journal of Food Protection 71 (2008) 2228–2232. doi:10.4315/0362-028x-71.11.2228.

[4] P. Brommesson, S. Sellman, L. Beck-Johnson, C. Hallman, D. Murrieta, C. T. Webb, R. S. Miller, K. Portacci, T. Lindström, Assessing intrastate shipments from interstate data and expert opinion, Royal Society Open Science 8 (2021). doi:10.1098/rsos.192042.

[5] T. Lindstrm, S. A. Sisson, S. S. Lewerin, U. Wennergren, Bayesian analysis of animal movements related to factors at herd and between herd levels: Implications for disease spread modeling, Preventive Veterinary Medicine 98 (2011) 230–242. URL: doi:https://doi.org/10.1016/j.prevetmed.2010.11.005.

[6] A. Gelman, J. B. Carlin, H. S. Stern, D. B. Rubin, Bayesian data analysis, Chapman & Hall, 2014.

[7] W. K. Hastings, Monte Carlo Sampling Methods Using Markov Chains and Their Applications, Biometrika 57 (1970) 97–109. doi:10.2307/2334940.

[8] P. H. Garthwaite, Y. Fan, S. A. Sisson, Adaptive Optimal Scaling of Metropolis-Hastings Algorithms Using the Robbins-Monro Process, arXiv:1006.3690 [stat] (2010). URL: `http://arxiv.org/abs/1006.3690`, arXiv: 1006.3690.

[9] G. O. Roberts, A. Gelman, W. R. Gilks, Weak convergence and optimal scaling of random walk Metropolis algorithms, The Annals of Applied Probability 7 (1997) 110–120. doi:10.1214/aoap/1034625254.

[10] S. P. Brooks, A. Gelman, General Methods for Monitoring Convergence of Iterative Simulations, Journal of Computational and Graphical Statistics 7 (1998) 434–455. URL: doi:10.1080/10618600.1998.10474787.
